# A scalable method for parameter-free simulation and validation of mechanistic cellular signal transduction network models

**DOI:** 10.1038/s41540-019-0120-5

**Published:** 2020-01-10

**Authors:** Jesper Romers, Sebastian Thieme, Ulrike Münzner, Marcus Krantz

**Affiliations:** 1 Institute for Biology, Humboldt-Universität zu Berlin, Berlin, Germany; 2 Bioinformatics Center, Institute for Chemical Research, Kyoto University, Uji, Japan

**Keywords:** Biochemical networks, Molecular biology, Software

## Abstract

The metabolic modelling community has established the gold standard for bottom-up systems biology with reconstruction, validation and simulation of mechanistic genome-scale models. Similar methods have not been established for signal transduction networks, where the representation of complexes and internal states leads to scalability issues in both model formulation and execution. While rule- and agent-based methods allow efficient model definition and execution, respectively, model parametrisation introduces an additional layer of uncertainty due to the sparsity of reliably measured parameters. Here, we present a scalable method for parameter-free simulation of mechanistic signal transduction networks. It is based on rxncon and uses a bipartite Boolean logic with separate update rules for reactions and states. Using two generic update rules, we enable translation of any rxncon model into a unique Boolean model, which can be used for network validation and simulation—allowing the prediction of system-level function directly from molecular mechanistic data. Through scalable model definition and simulation, and the independence of quantitative parameters, it opens up for simulation and validation of mechanistic genome-scale models of signal transduction networks.

## Modelling: signal transduction at the genome scale

Mechanistic models are like road maps—they help us understand how signals travel through cells and guide cellular decisions. We developed a method to turn these models executable at the genome scale, and show that this method can explain and predict cellular physiology directly from molecular mechanisms. The bottom line is that the regulatory structure of the network is encoded in the molecular mechanisms, and we capture this knowledge in two generic update rules. These can be used to assemble molecular mechanisms, LEGO-brick style, into a functional model—without parametrisation or model fitting. This makes it possible to use the method for network validation and simulation of systems that are far too large to be parametrised, opening the door to genome-scale mechanistic models of cellular signal transduction.

## Introduction

Systems biology aims at the integrative analysis of large-scale biological systems up to whole cells. To realise this goal, we integrate knowledge into executable or computational models.^1^ This process has been developed the furthest in the field of metabolic modelling, where the community routinely works with genome-scale models. These models are defined at the level of biochemical reactions, cover the entire metabolic network of even complex cells, and can be simulated to predict system-level functionality.^2,3^ The methodology is well established and supported by rich toolboxes for network reconstruction, validation and simulation,^4^ and it constitutes the paradigm for bottom-up modelling. However, these tools cannot be used for signal transduction networks, due to the difference between mass and information transfer networks.^5^

Mechanistic modelling of signal transduction is challenging at several levels. First, at the level of model definition: empirical data on site-specific modifications of, or bonds between, signalling components combine combinatorially into a large number of possible configurations, or microstates.^6^ This combinatorial complexity effectively makes it impossible to create detailed mechanistic models of large signalling networks using models based on enumeration of microstates (as in e.g. normal ODE-models, Petri nets or SBGN-PD diagrams).^7^ Consequently, representation and simulation of these networks were limited to very small and/or heavily simplified models. To solve this, the community developed simulation tools with adaptive resolution, such as the rule-based modelling languages BioNetGen and Kappa.^8,9^ Second, simulation may be prohibitively expensive even with an efficient model definition, as is the case for classical rule-based modelling, in which the full network of microstates must be generated. This was solved by the development of the network-free simulation tool for rule-based models, NFsim.^10^ Third, quantitative dynamic models require rate laws that must be parametrised in terms of rate constants and initial molecule amounts. Reliable information on these quantities is sparse, precluding meaningful parametrisation of most mechanistic models. While the lack of quantitative knowledge is an experimental rather than a theoretical challenge (more dedicated biochemistry is required), a simulation method that voids the need for parametrisation would be extremely helpful in evaluating mechanistic large-scale signalling models until this knowledge gap can be filled.

Parameter-free simulation of cellular networks is typically performed through constraint-based or Boolean methods.^11^ Constraint-based methods rely on mass transfer *through* the network, and can therefore not be used to model signal transduction which primarily rely on interaction between and reversible modification of the signalling components. In contrast, Boolean methods can be used to simulate signal transduction networks. In these networks, entities (called “targets” below)—typically representing signalling components—are either true (active) or false (inactive) and edges define how the target states at time *t* + 1 depend on the target states at time *t*. Boolean networks have been used extensively in modelling, and different methods and toolboxes have been developed.^12^ However, most of these Boolean models are not *mechanistic*, i.e. they abstract signalling to a binary on/off state of components, ignoring the actual state changes that transmit the information. While such phenomenological modelling have been used to simulate signalling successfully, models that account for the mechanisms of signal transduction increase the explanatory and predictive power by: (i) accounting for signalling components with more than two (on/off) states, such as the cyclin-dependent kinase that are activated against different targets by different cyclins. (ii) Enabling the model to have the same resolution as data, facilitating model creation and evaluation as well as comparison to empirical data. (iii) Supporting functional analysis as e.g. in simulation of the effect of (combinations of) point mutations. Despite the potential advantages, only three mechanistic Boolean modelling methods have been developed until today: one is derived from SBGN-PD diagrams, one from rule-based models, and one is based on rxncon, the reaction-contingency language.^13–15^ These methods support a detailed description of signalling events. However, the first is based on a microstate description, inheriting the scalability issues of these approaches;^13^ the second requires a fully parametrised rule-based model, inheriting the problem of parametrisation;^14^ and the third inherited shortcomings in expressiveness and precision from the first generation of the rxncon language.^15^ Consequently, new methods are needed to support parameter-free, and hence scalable, simulation of signal transduction networks.

**Fig. 1:**
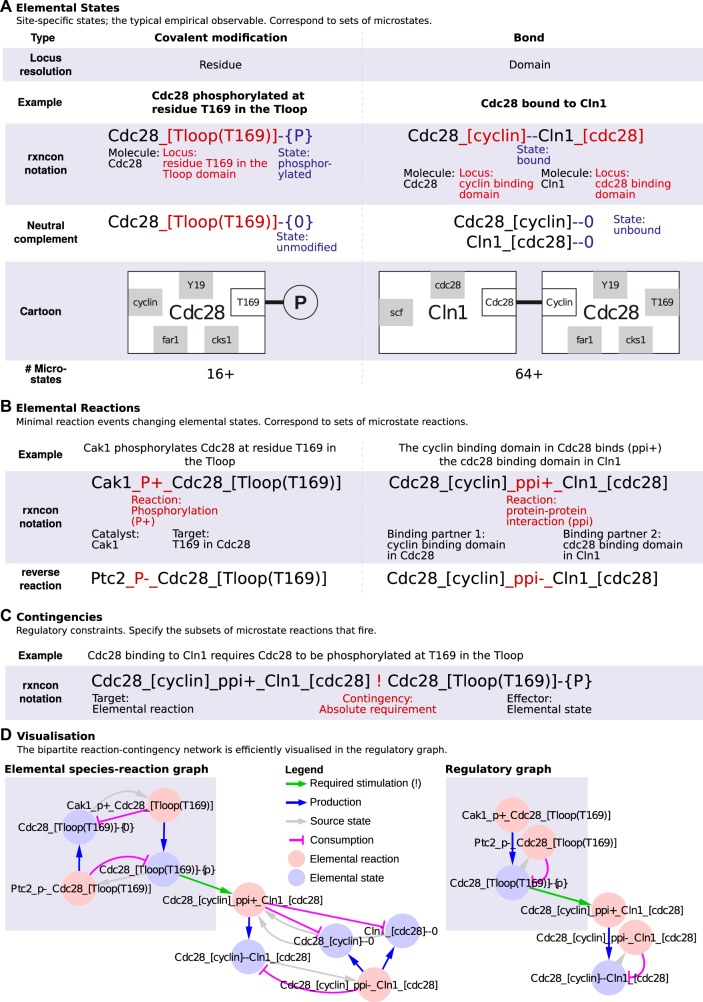
The reaction-contingency (rxncon) language employs three essential components. Elemental states, elemental reactions, and contingencies. The regulatory graph efficiently represents the regulatory structure of the network. **a** Elemental states are empirical observables: signalling molecules encode information through site-specific state changes, i.e. *covalent modification* of specific *residues* or *bonds* between specific *domains*. All elemental states at a single locus are mutually exclusive with each other and their neutral complements (unmodified and unbound, respectively). However, each elemental state only defines the state at a single locus (two for bonds; cartoon: white boxes), leading to a one-to-many relationship between the (empirically measured) elemental states and the (inferred) microstates. **b** Elemental reactions are indivisible reaction events defined in terms of elemental states. They are only defined in terms of the elemental states that are produced, consumed, synthesised or degraded, similar to the reaction centre of a rule in RBMs, and hence have a one-to-many relationship to microstate reactions. **c** The contingencies define constraints on an elemental reaction in terms of elemental states (or Inputs) that do not change through the reaction. Hence, the contingencies reduce the number of valid microstate reactions, and correspond to the reaction contexts of RBMs. Boolean contingencies, employing AND, OR and NOT gates, can specify arbitrarily detailed constraints down to microstates as necessary. Due to this adaptive resolution, rxncon models can faithfully mirror the empirical knowledge. **d** The regulatory graph (right) provides a compact representation of the regulatory structure of the rxncon network. It is a simplified version of the elemental species-reaction graph (left), leaving out the neutral states to reduce graph complexity, to remove non-informative cycles, and to improve readability. Both graphs are bipartite, with two types of nodes and two types of edges: reaction-to-state (reaction) edges show the effect of each reaction on its source and product states, and state-to-reaction (contingency and source state) edges the impact of states on reactions. The bipartite nature of the graph highlights the requirement for both reactions and contingencies for information transfer through the network, as both types of edges are necessary to traverse the graph. This figure and legend is reproduced from ref. ^17^.

Here, we present a parameter-free simulation method that supports large-scale mechanistic models of signal transduction networks. This bipartite Boolean modelling (bBM) logic is based on the second generation rxncon language (Fig. 1), which is tailored for formalising signal transduction models based on empirical data:^16^ the reaction network is defined in terms of *elemental states*, i.e. modifications (or lack thereof) at specific residues and bonds (or lack thereof) at specific domains. The definition is bipartite: *elemental reactions* are decontextualised reaction events that define how elemental states are *synthesised, degraded, produced*, or *consumed*, and *contingencies* define how elemental reactions depend on (combinations of) elemental states that are not changed through the reaction. This structure is kept in the bBM: the *state targets* track which components are present, and in which states the components are (i.e., which domains are (not) bound to which other components and which residues are (un)modified). The *reaction targets* track which reactions are eligible to fire. Through a constructive approach, we define two generic update rules for these two target types, and demonstrated that the update rules can be assembled, LEGO-brick style, into large-scale models without any need for optimisation at the system level. Furthermore, we show that the resulting models meaningfully reproduce the behaviour of signalling processes, and that discrepancies between model behaviour and system-level expectations can be used to identify gaps in the network reconstruction and hence to improve the model. In a parallel effort, we use the rxncon language and the bBM formalism presented here to build and analyse a comprehensive and mechanistically detailed model of the network that controls the cell division cycle in baker's yeast^17^—validating both the scalability as well as the explanatory and predictive power of the method. Taken together, we present a scalable method for parameter-free validation of mechanistic signal transduction network models, taking an important step to close the gap in capabilities between metabolic and signal transduction modelling, and introduce a method for scalable simulation of signal transduction networks that supports modelling at the genome scale.

## Results

### A new approach to Boolean modelling

The central result presented in this work is a method to derive a parameter-free Boolean model from a rxncon network, in a completely mechanistic fashion. The starting point is an arbitrary model defined in the second generation rxncon language, and the end point a bipartite Boolean model prepared for simulation with the Boolnet package.^18^ The model generation process is implemented in the rxncon compiler, which generates three output files: A.boolnet file containing the model, a.csv file containing the initial values of all targets (see discussion below) and a.csv file linking the model aliases to the original rxncon names. The two first are used to simulate the model, and the third is necessary to interpret the results (see the methods section for detail). Hence, we use a well-established format for Boolean modelling, but the meaning we assign to nodes and edges are fundamentally different than those used in previous Boolean models. Below, we describe the derivation of this modelling logic and exemplify it on two minimal motifs, a small pathway and a detailed model of the yeast pheromone pathway that cannot be meaningfully simulated with conventional methods at this level of mechanistic resolution.

### A rxncon model uniquely defines a bipartite network

A rxncon model can be represented by a bipartite network (elemental species-reaction graph, see Fig. 1), which consists of two distinct types of nodes: *elemental states* and *elemental reactions*. These nodes are connected by two distinct types of directed edges: *reaction edges*, which connect elemental reactions to elemental states, and *contingency edges*, which connect elemental states to elemental reactions. The mapping from a rxncon network to an elemental species-reaction graph is unique: the reaction list of the rxncon model contains the information needed to generate the nodes and the reaction edges, i.e. which elemental reactions and states that exist, which elemental reactions change which elemental states, and how. We consider two types of elemental states; *internal states*, which are the property of a single molecule (e.g. covalent modifications such as phosphorylation or ubiquitylation, or an empty binding domain) and *bond states*, which are shared between two molecules or two domains in the same molecule (e.g. protein-protein interactions and intra-protein interactions, respectively). Importantly, both these types of states are *elemental*, meaning they define a single, indivisible property of one molecule (two for intra-molecular bonds), at a single site (two for bonds) which we call residue (for modifications) or domain (for bonds). An elemental reaction can act on each state in one of four different ways: through *synthesis* (the state appears together with the component as the latter is synthesised), *degradation* (the state disappears together with the component as the latter is degraded), *production* (the state appears on an already present component) and *consumption* (the state disappears but the component remains). The mode of action is defined from the perspective of each state. For example, the Cak1_P + _Cdc28_[T169] phosphorylation reaction produces the phosphorylated Cdc28_[(T169)]-{P} state and consumes the unphosphorylated Cdc28_[(T169)]-{0} state (Fig. 1a, b). The effect of a reaction is directly defined by the *skeleton rule* underlying the reaction.^16^ This rule is similar to a rule-based model rule, such as can be defined in BNGL, but consists solely of a centre, without context:

The definitions, where RHS and LHS refer to the right-hand respectively left-hand side of the skeleton rule, are: *Production:* a state is produced by a reaction if it appears on the RHS, not on the LHS, but the component carrying the state does appear on the LHS. *Consumption:* a state is consumed by a reaction if it appears on the LHS, not on the RHS, but the component carrying the state does appear in the RHS. *Synthesis:* a state is synthesised by a reaction if it appears on the RHS, and the component carrying the state does not appear on the LHS. *Degradation:* a state is degraded by a reaction if the component carrying the state appears on the LHS, no state mutually exclusive with it appears on the LHS, and the component carrying the state does not appear on the RHS.

The contingency list contains the information needed to generate the contingency edges, i.e. which elemental states influence which elemental reaction, and how. This information corresponds to the reaction context of rule based models, i.e. they define the contextual constraints on the reactions in terms of elemental states that do not change through the reaction. There are six types of contingencies that define which role an elemental state plays in a reaction: absolutely required (“!”), stimulating (“K+”), no effect (“0”), inhibitory (“K−”), absolutely inhibitory (“x”), and no known effect (“?”; treated as “0”). The qualitative contingencies (“!”/“x”) must be fulfilled for a reaction to occur, while the quantitative contingencies (“K+”/“K−”) change the rate with which a reaction occurs. Of course, many reactions depend on more than one elemental state, such as reactions requiring dual phosphorylation or the assembly of a multimeric complex. Such complex contingencies that require negation of and/or multiple elemental states can be defined using Boolean (AND, OR, NOT) contingencies, which, for clarity, are visualised as separate nodes in the graph.

*Note*: *The resulting graph is deceptively similar to the bipartite species-reaction graphs used in e.g. metabolic networks or SBGN-PD diagrams. However, the elemental states are not disjunct—i.e., a single molecule typically occupies several elemental states. In fact, each molecule must occupy exactly one elemental state for each residue and domain that the molecule contains (see* Fig. 1a*). In contrast, SBGN-PD, the KEGG metabolic maps or Petri nets uses disjunct microstates that are mutually exclusive. Microstates are defined by a combinatorial enumeration of all (relevant) elemental states and therefore associated with severe scalability issues. In these, each molecule occupies exactly one microstate. This critical difference precludes the use of simulation methods developed for disjunct networks, and necessitates the development of a new simulation method tailored to rxncon network models*.

### From a rxncon network to a Boolean model: basic assumptions and desired model behaviour

The first step in defining the modelling logic is to define what true and false means for the elemental reaction and state targets. The interpretation we assign to the value of these targets is as follows:

- If a *reaction target* is true, the cellular regulatory network is, at that point in time, in a configuration where it can accommodate that reaction. This is required, but not sufficient, for the reaction to take place: In the absence of its source state(s), a reaction will not “fire” even though its value is true, as the reaction targets are purely a description of the regulatory layer of the biological cell. In rxncon terms, the reaction updates implement the contingencies.
- A *state target* is true if there are a sufficient number of molecules carrying that state present in the cell for it to be considered functionally relevant. In rxncon terms, the state updates implement the elemental reactions.

The second step is to define the desired model behaviour. We desire both reaction and state targets to describe *system-level* properties, not molecular ones. A consequence of this is, for example, that two states that are mutually exclusive on a single molecule (e.g. a single residue being phosphorylated and unphosphorylated), can be simultaneously true in our Boolean system.

The behaviour of the reaction targets is determined by the contingencies. The basic interpretation is straightforward: reactions should only fire if all required (“!”) and no absolutely inhibitory (“x”) contingencies are fulfilled. We note that quantitative effects (“K+”/“K−”) lack interpretation in a deterministic Boolean system, and chose to treat them as no effect (“0”), but also implement the option to treat them as absolutely required or inhibitory, respectively. To accommodate contingencies consisting of multiple elemental states we need to make our first assumption: that any combination of elemental states is true if all single elemental states are true. With this assumption, it becomes straightforward that for a reaction to be true, all required elemental states (“!”) must be true and all absolutely inhibitory elemental states (“x”) must be false.

The state targets are determined by the reactions. Here, we assume a quasi-steady-state at each update step. From this follows a natural hierarchy between the reaction types introduced above: *synthesis* is stronger than *degradation* which is stronger than *production* which is stronger than *consumption*. Due to the crude concept of Boolean time, we consider a quasi-steady-state at each update step. It is then easy to see that a protein that is both synthesised and degraded must be present in the system. In the case where the synthesis reaction is too weak to maintain functional level of the protein, it would be considered off. Similar, production, from the perspective of a specific state, will be dominant over consumption: in the presence of a phosphorylation cycle with both kinases and phosphatases active, both forms will be present. Finally, degradation is dominant over production, as depletion of a protein will deplete the phosphorylated form regardless of kinase activity (except when protected from degradation, as covered by contingencies).

**Fig. 2:**
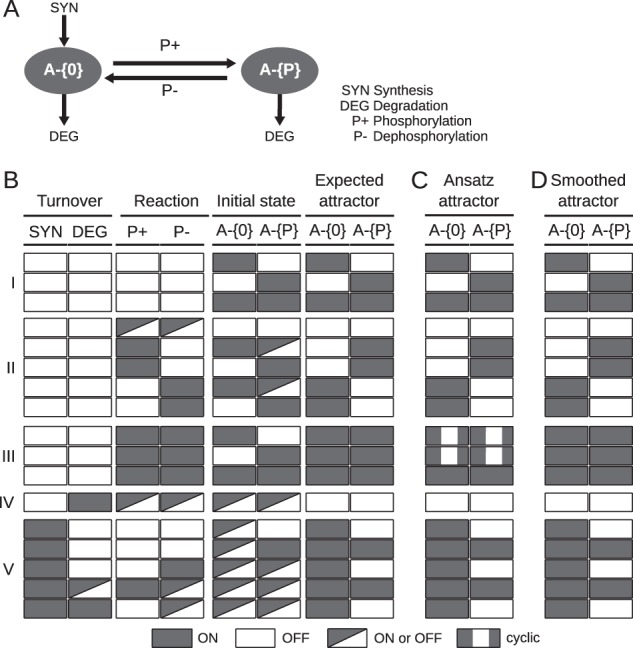
Behaviour of a minimal modification motif. **a** The motif includes two states of A, unphosphorylated (A-{0}) and phosphorylated (A-{P}). The motif contains four different reaction types: component A can be synthesised (in its neutral state A-{0}), degraded (in either state), phosphorylated (consumes A-{0}, produces A-{P}) or dephosphorylated (consumes A-{P}, produces A-{0}). The regulatory and elemental species-reaction graphs of the motif can be found in Supplementary Fig. 1. **b–d** Initial conditions, expectations and simulation results. Each line correspond to the simulation of one or more model variants, and visualise which reactions are active, which states are initiated, which behaviour we expect, and the behaviour we observed. **b** To define the desired behaviour of the motif, we create 64 variants of the motif with each of the four reactions constitutively ON (true) or OFF (false) (columns 1–4), and each of the elemental states initially true or false (columns 5 and 6), and define the expected steady state as a function of initial state and active reactions (“Expected attractor”; columns 7 and 8). (I) In the absence of any reactions, the steady state will be identical to the initial state. (II–III) In the absence of synthesis or degradation, but in the presence of component A (A-{0} or A-{P} true), the equilibrium depends on the (de)phosphorylation reactions. With only one of these reactions, only the fully (de)phosphorylated form is present at steady state. However, with both reactions present we expect both elemental states to be present at steady state. (IV) With degradation but not synthesis active, the protein will be depleted and both states will be false at steady state. (V) With active synthesis, the neutral state will always be present. The phosphorylated state will only be present if there is also a phosphorylation reaction or if the state is initially present and both the degradation and dephosphorylation reactions are off. **c** The attractors reached after simulation with the update rules in the original ansatz. The attractors correspond to the expected attractors (see B) for 62 out of 64 configurations. The exception are the cyclic attractors in block (III), where both phosphorylation and dephosphorylation are active and only one of the elemental states are initiated as true. **d** The attractors reached after simulation with the smoothed update rules. The attractors are identical to the expected attractors in all cases.

**Fig. 3:**
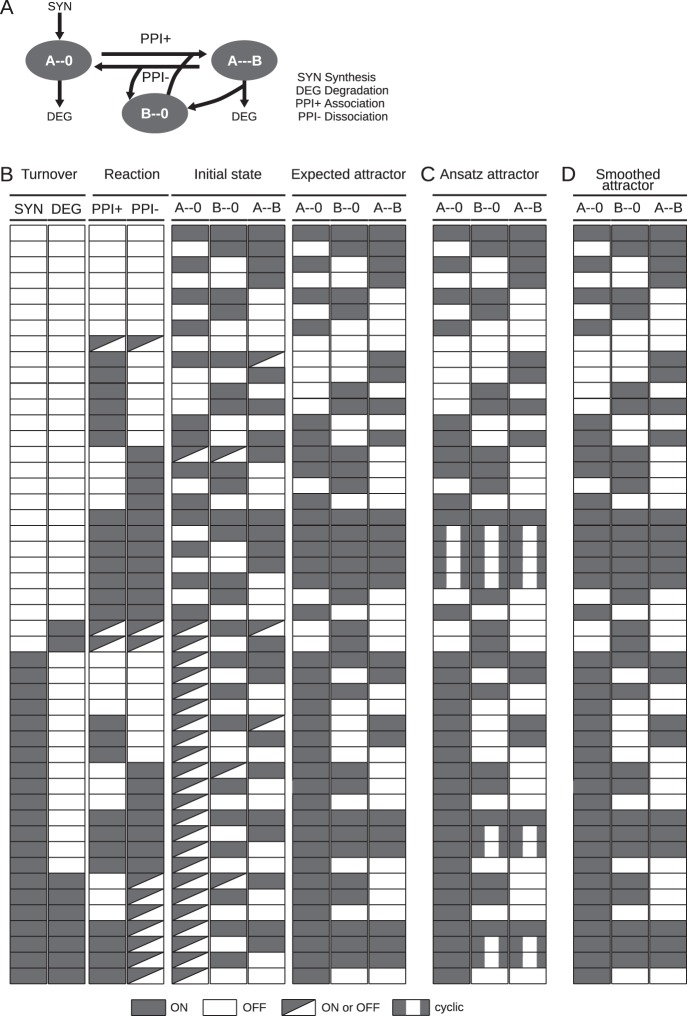
Behaviour of a minimal interaction motif. **a** The motif includes the unbound state of A (A--0), the unbound state of B (B--0) and the bound state (A--B). As in Fig. 2, the motif contains four different reaction types: component A can be synthesised (in its neutral state A--0) or degraded (in either state), and Component A and Component B can bind (ppi+; consumes A--0 and B--0, produces A--B) or dissociate (ppi- consumes A--B, produces A--0 and B--0). Not that degradation of A in the A--B dimer releases B--0, hence this reaction is a degradation reaction of A and conditional production reaction for B--0. The regulatory and elemental species-reaction graphs of the motif can be found in Supplementary Fig. 1. **b–d** Initial conditions, expectations and simulation results. Each line correspond to the simulation of one or more model variants, and visualise which reactions are active, which states are initiated, which behaviour we expect, and the behaviour we observed. **b** To define the desired behaviour of the motif, we create 128 variants of the motif with each of the four reactions constitutively ON (true) or OFF (false) (columns 1–4), and each of the elemental states initially true or false (columns 5–7), and define the expected steady state as a function of initial state and active reactions (“Expected attractor”; columns 8 and 9). As in Fig. 2, the initial state is preserved when no reaction is active. However, the steady state in the presence of active reactions is more complex, as both unbound states are necessary for the reaction to fire—which affects both the generation of the A--B state and the depletion of the unbound states. With degradation and without synthesis, A--0 and A--B is removed, releasing B--0 in the latter case. Finally, synthesis of A only leads to A--B in the presence of the forward reaction, in which case B--0 is depleted unless A--B is turned over by degradation or dissociation. **c** The attractors reached after simulation with the update rules in the original ansatz. The attractors correspond to the expected attractors (see B), except when either component is present in only one state (unbound or bound). This happens in the analogous case to the spurious oscillations in the modification motif in Fig. 2, but also when A is synthesised if B is only present in one form. **d** The attractors reached after simulation with the smoothed update rules. The attractors are identical to the expected attractors in all cases.

To define the desired behaviour in detail, we designed and studied the minimal irreducible motifs containing two (for modification reactions; Fig. 2a) or three elemental states (for interactions; Fig. 3a), and the reactions acting upon them. Each motif contains four reaction types that synthesise, degrade, produce or consume the states. By varying which reactions are active and inactive, and which states are initially present, we arrive at 64 (2^6^) and 128 (2^7^), respectively, distinct models for the two motifs. We define, given an initial configuration for the states and the absence or presence of each of the reactions, the desired steady-state behaviour (Figs. 2b and 3b). The crux of the matter then becomes to find update rules for the states so that the (deterministic) attractor reproduces this steady state.

### Defining the notation: reactions, states and components

A rxncon system will contain *N*_*R*_ reactions denoted by *R*_*i*_, *N*_*s*_ elemental states denoted by *S*_*i*_, and *N*_*c*_ components denoted by *C*_*i*_. For the components that appear without any internal states, such as e.g. those that solely appear as catalysts, the component is its own state (which does not appear in the original rxncon system). For those components that do carry internal or bond states, the component can be expanded as a Boolean expression of elemental states grouped by the site (domain or residue) on which they live (Eq. 1):

1 $$C_i = \mathop {\bigcap}\limits_{\begin{array}{*{20}{c}} {{\rm{site}}\,j\,{\rm{on}}} \\ {{\rm{component}}\,i} \end{array}} {\mathop {\bigcup}\limits_{\begin{array}{*{20}{c}} {{\rm{state}}\,k\,{\rm{on}}} \\ {{\rm{site}}\,j} \end{array}} {S_k} }$$

The origin of this expression is in the mutual exclusivity of elemental states that live on the same residue or domain: for each of these sites, at least one of the states living on the site needs to be present for the component itself to be present. A consequence of this is that for components that carry internal or bond states, the dynamic behaviour of the component is fully determined by the states which the component carries.

Furthermore, we define functions mapping states and reactions to Boolean expressions of states. First, the functions *K*(*R*_*i*_) list the components which are reacting in reaction *R*_*i*_ (Eq. 2):

2 $$K\left( {R_i} \right) = \mathop {\bigcap}\limits_{\begin{array}{*{20}{c}} {{\rm{component}}\,j} \\ {{\rm{reacts}}\,{\rm{in}}\,R_i} \end{array}} {C_j}$$

whereas the functions *K*(*S*_*i*_) list the components carrying the state *S*_*i*_. Bond states are carried by two components, whereas modifications are carried by one (Eq. 3):

3 $$K\left( {S_i} \right) = \mathop {\bigcap}\limits_{\begin{array}{*{20}{c}} {{\rm{component}}\,j} \\ {{\rm{carries}}\,S_i} \end{array}} {C_j}$$

In the update rule for the states, we will require the following combinations. First, a reaction together with its source states (Eq. 4):

4 $$R_i^\prime = R_i\mathop {\bigcap}\limits_{\begin{array}{*{20}{c}} {S_j\,{\rm{consumed}}} \\ {{\rm{by}}\,R_i} \end{array}} {S_j}$$

The neutral state (unmodified, unbound) counterpart for a particular state *S*_*i*_ is denoted by *N*(*S*_*i*_).

These notations enable us to write down the synthesis term Σ, which describes whether a state is either directly or indirectly being synthesised (Eq. 5):

5 $${\mathrm{\Sigma }}\left( {S_i} \right) = \left\{ {\begin{array}{*{20}{l}}\qquad\qquad {\mathop {\bigcup}\limits_{\begin{array}{*{20}{l}}{R_j\,{\rm{synthesizes}}} \\\qquad {S_i} \end{array}} {R_j^\prime } \,{\mathrm{for}}\,{\mathrm{neutral}}\,{\mathrm{states}}\,S_i} \hfill \\ {\mathop {\bigcup}\limits_{\begin{array}{*{20}{l}} {R_j\,{\rm{synthesizes}}} \\ {N(S_i)} \end{array}} {R_j^\prime } \bigcap \mathop {\bigcup}\limits_{\begin{array}{*{20}{c}} {R_k\,{\rm{produces}}} \\ {S_i} \end{array}} {R_k^\prime } \,{\mathrm{for}}\,{\mathrm{non}}{\text{-}}{\mathrm{neutral}}\,{\mathrm{states}}\,S_i} \hfill \end{array}} \right.$$

All systems we considered contain only states “one step” removed from the neutral states, so the expression for non-neutral states describes an active path coming from the synthesised state to the state under consideration. For states that are multiple steps removed from the synthesised neutral state, this expression has to be appropriately amended.

Finally, we denote the Boolean expression representing the contingency for reaction *R*_*i*_ by *L*(*R*_*i*_) (Eq. 6):

6 $$L\left( {R_i} \right) = \mathop {\bigcap}\limits_j {L_j^!} (R_i)\mathop {\bigcap}\limits_k {\overline {L_k^ \times (R_i)} }$$

where the $L_j^!(R_i)$ and $L_k^x(R_i)$ enumerate the required, respectively, inhibitory contingencies for reaction *R*_*i*_, which are themselves possibly nested Boolean expressions.

#### The expected behaviour of a small reaction circuit and update rule ansatz

The reaction update rules are quite straightforward. We require the strict contingencies for the reaction to be satisfied and the presence of the components on which the reaction acts (Eq. 7):

7 $$R_i\left( {t + 1} \right) = K(R_i;t) \cap L(R_i;t)$$

The state update rules are more complex and explicitly require the hierarchy between types of reactions that was alluded to above. First of all, if a state is synthesised by any reaction, it will be true. If synthesis is false, the necessary, but not sufficient, requirement for the state to be true is that degradation is also false, and that the component(s) carrying the state are present. Now, there are two options for the state to be true: either the state is being produced by some reaction, in which case it is immaterial what the previous value of the state was, or the state was already true and it is not being consumed by any reaction (Figs. 2 and 3). Eq. 8:

8 $$S_i\left( {t + 1} \right) = {\mathrm{\Sigma }}(t) \bigcup \left( {K(S_i;t)\mathop {\bigcap}\limits_{\begin{array}{*{20}{c}} {R_k{\rm{degrades}}} \\ {S_i} \end{array}} {\overline {R_k^\prime } (t)} \bigcap \left\{ {\mathop {\bigcap}\limits_{\begin{array}{*{20}{c}} {R_l\,{\rm{produces}}} \\ {S_i} \end{array}} {{R_l^\prime } (t)} \bigcup \left[ {S_i(t)\mathop {\bigcap}\limits_{\begin{array}{*{20}{c}} {R_m{\rm{consumes}}} \\ {S_i} \end{array}} {\overline {R_m^\prime } (t)} } \right]} \right\}} \right)$$

In other words, a state remains true as long as it is neither consumed nor degraded. If a reaction produces the state, it becomes or remains true even when it is actively consumed, but not if it is degraded. Finally, if the state is synthesised, it becomes or remains true regardless of any other active reaction. See supplementary discussion for an example.

As can be seen in this formula, the translation of “the state is being produced” *et cetera* contains the primed reactions, meaning the reaction producing that state and the source state(s) of that reaction (see Eq. 4). This is due to the semantics of the reaction targets, which only tell us about the regulatory state of the network.

Note that most reactions act on multiple states (typically source and product states). It is imperative that these are updated simultaneously to avoid simulation artefacts. Hence, we use only synchronous deterministic update schemes.

### Testing the generic update rules

To test if the update logic described above captures the expected behaviour, we implemented the model generation process and used it to generate the 64 models corresponding to the minimal modification circuitry above (Fig. 2). The models were simulated using BoolNet,^18^ as described in the methods. The attractor states are visualised in Fig. 2c. The model behaviours correspond to our expectations with one notable exception. In the absence of synthesis and degradation, but in the presence of both phosphorylation and dephosphorylation, the model displays an oscillatory behaviour when only one of the two states is initiated. Closer inspection reveals that this is due to periodic source state depletion. The phosphorylation and dephosphorylation reactions are constitutive (no contingencies, no loss of components), and the oscillations occur due to the state updates, which are completely encoded in the state update rule. Indeed, as soon as a reaction executes, dependent on its source state, it depletes the source state pool. Hence, the reactions alternate in firing, triggering out-of-phase oscillations in the truth value of the states. Consistently, these oscillations disappear when both states are initiated or when the source state is repleted through synthesis. We observe the same phenomenon in the interaction motif, when the reaction cycle is active and at least one component is initiated in and remains in a single form (Fig. 3c). We consider these spurious oscillations undesirable in our systems-level description, but note that they would be appropriate for models of single molecules. Nevertheless, the outcome is highly encouraging, as 62 out of 64 models matched the expected behaviour.

### Source state smoothing eliminates the spurious oscillations

To eliminate the oscillations that plagued our initial ansatz, we adapted the updates rules for states by widening the window in which we checked for source state availability: a reaction needs the source state to be present, or to be produced. To achieve this, in Eq. (8) we substitute for the reactions producing the state, *R’*_*l*_ the following (Eq. 9):

9 $$R_l^{\prime\prime} (t) = R_l(t)\mathop {\bigcap}\limits_{\begin{array}{*{20}{c}} {S_j\,{\rm{consumed}}} \\ {{\rm{by}}\,R_l} \end{array}} {S_j(t)}\,{\bigcup}\, {S_j(t + 1)}$$

where the S_j_(t + 1) is the full expression (Eq. 8), without this substitution.

We consider this adaptation quite natural: there are large numbers of molecules undergoing the same set of reactions. Therefore, it is highly unlikely that all of these reactions are temporally completely in phase, justifying a smoothing over molecules. In addition, the time scale in a Boolean model is basically set by the slowest of the reactions, since all rate constants are absent. For molecule pools acted upon by reactions that are faster than the slowest in the system, it is likely that they will pass through mutually exclusive states within the window of a Boolean time step, justifying a “time smoothing”. Both effects are captured by the reworked update rules.

The smoothing assumption only breaks down in the context of few molecules and low reaction rates, and there are cases in which smoothing is inappropriate. In other work,^17^ we have introduced a hybrid model containing both the molecular reactions and states described here, and additionally macroscopic reactions and states that are governed by the non-smoothed update rules. However, for most states in a signal transduction network, the smoothed update rule is more appropriate. Having established the smoothing logic, we implemented it into the rxncon compiler tool and recreated the 64 modification motif (Fig. 2) and 128 interaction motif (Fig. 3) models with smoothing. We repeated the simulation and compared the results to the original simulation (Fig. 2d, Fig. 3d). The oscillatory behaviour disappeared, but no other simulation results changed. Hence, the simulation results exactly match the behaviour we expect from a model of these reaction motifs.

### The update rules can be used as LEGO bricks to assemble a systems level model

**Fig. 4:**
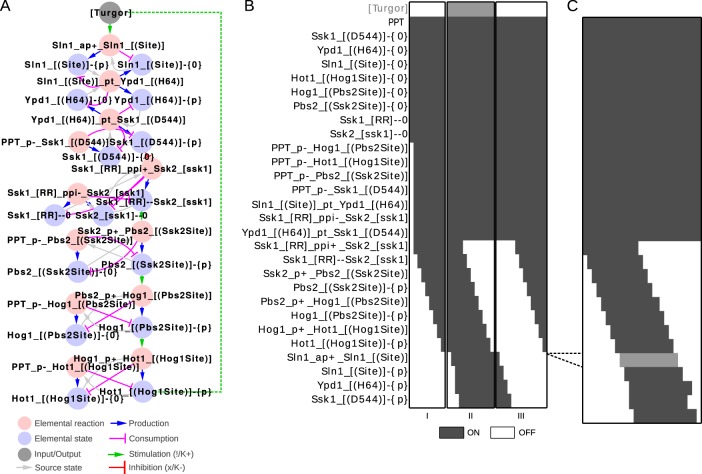
From reactions to a functional pathway. We used the smoothed update rules to generate and simulate a model of the Sln1 branch of the high osmolarity glycerol (HOG) pathway. **a** The pathway visualised as a rxncon regulatory graph. In the absence of turgor, Sln1 stays unphosphorylated. As turgor increases, the auto-phosphorylation of Sln1 initiates a phosphotransfer cascade converging at Ssk1. The phosphorylated form of Ssk1 turns off the downstream MAP kinase pathway leading to dephosphorylation of the downstream transcription factor Hot1. The dashed line indicates a feedback loop that is included in the cyclic version only. The model has 29 different targets: 12 reaction targets (pink nodes), 16 state targets corresponding to 15 elemental states (blue nodes) and a component state for the phosphatase (not displayed in the graph), and the single input/output target “Turgor” (grey node). **b** Simulation of a linear version of the model using source state smoothing of the update rules. (I) We use our default assumptions on the initial state and simulate the model until we reach an attractor (first OFF trajectory). (II) We activate the system by turning [Turgor] ON and simulate again (ON trajectory) until we reach an attractor state. (III) From there, we set [Turgor] OFF again and simulate the model until we reach an attractor. We observe that the model responds as expected to the input. **c** We extended the HOG model with a feedback loop, where activation of the pathway leads to increased turgor (via Hot1-{P}). This is simplification of an adaptive response through increased glycerol production and retention, which increases turgor. We simulate this model from the initial OFF attractor (see panel B), and note that the system oscillates as expected: the trajectory is cyclic, where the last time step is followed by the first, and turgor is now a model variable that turns on and off during the cycle (grey row in the heatmap).

**Fig. 5:**
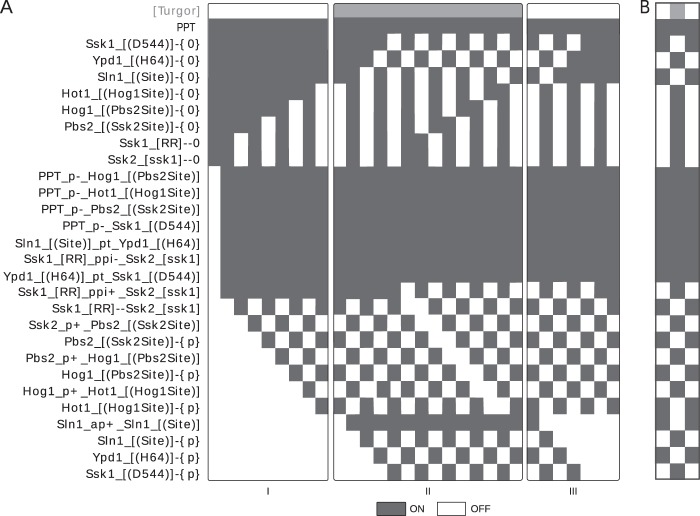
Smoothing is required to make pathway simulation interpretable. We repeated the simulation of the HOG pathway with the un-smoothed update rules from the initial ansatz. **a** Simulation of the linear model without smoothing. The signal goes through the pathway, but analysis is complicated by the spurious oscillations as the system no longer converges on point attractors. Each of the three simulation trajectories (I-III) ends with a cyclic attractor of length two. **b** Simulation of the cyclic HOG model without smoothing. Here, the entire oscillation cycle breaks down into a period two oscillator involving all the states (and their complements) and reactions that transmit the information.

Next, we applied the bBM logic to simulate a linear pathway. Both the reaction and contingency motifs are fully defined at the local level, as they only depend on the reactions (for state updates) and the contingencies (for reaction updates) in the system. We postulated that these locally defined update rules would suffice to define system-level function, and tested this hypothesis with a simplified model of the HOG MAP kinase pathway from *Saccharomyces cerevisiae* (Fig. 4; adapted from^15^). We created a rxncon 2.0 model of this pathway (Supplementary Model 1), and used this to generate the bBM using the generic update rules with smoothing (see Supplementary discussion for implementation details). Already this small model has 28 reaction and state, and hence 2^28^ (~10^8^) possible initial states. We deemed this too many for an exhaustive search, and decided to use a generic start state for all simulations: all neutral elemental states (that is, unbound binding domains and neutral modifications) are true, all generic component states (for components with no elemental states) are true, and all other targets are false. From this highly artificial initial state, we let the model find its own natural “off state” by executing it until an attractor is reached (Fig. 4b). At this point, we take this attractor as a new initial state and change the input state (Turgor in this case) and repeat to see the response of the pathway to the input, and repeat this process until the model returns to a state vector we have already seen. As can be seen from Fig. 4b, the HOG pathway responds appropriately to turgor: it turns off the kinase cascade. For comparison, we repeated the simulation with the non-smoothed logic (Fig. 5a), where we see the signal passing through the network despite spurious oscillations. However, the system does not converge to point attractors, leading to more complex analysis and interpretation. There are three striking blocks in the heatmap (Fig. 4b): first, the initial neutral states never turn off. Second, there is a block of reactions that turns on directly, and stays on throughout the simulation. Third, there is a block that turns on and off in response to the signal. The third block contains the reactions and states that actually transmit the information. The second block contains constitutive reactions, which are either unregulated (e.g. dephosphorylation reactions), or regulated at the level of source state availability (e.g. phosphotransfer from Sln1 to Ypd1). The first block contains all the neutral states. These remain true because the reactions that produce them are considered unregulated, which may be due to experimental bias as discussed below. Hence, the logic of the generic update rules is sufficient to convert the molecular level knowledge in a rxncon network into a functional bBM that accurately predicts system-level function. It is highly non-trivial that generic update rules, which were defined for isolated reactions and states, suffice to define a complete model that functions at the systems level, with no further tweaking or parametrisation. Taken together, the generic update rules map a given rxncon network on a unique Boolean model that predicts systems level function.

### The bipartite Boolean logic correctly reproduces real oscillations

The HOG pathway is a homeostatic pathway that maintains proper turgor pressure. The pathway output eventually leads to signal cessation through a physiological feedback loop.^19^ To simulate this, we linked the most downstream component to the input that turns the pathway off (dashed line in Fig. 4a). We repeated the model creation and simulation, using the initial steady state of the linear model as starting condition. As shown in Fig. 4c, the model now shows a periodic activation/deactivation behaviour, similar to that when the input is changed manually. Hence, the bBM logic is fully capable of predicting biologically relevant oscillations. As comparison, we also simulated the cyclic HOG model without source state smoothing (Fig. 5b). Here, the pathway signal is completely washed out by the spurious oscillations and breaks down to a two-state cyclic attractor. The source state smoothing facilitates bBM analysis and clearly improves the interpretability of the simulation results. Taken together, the bBM logic generates Boolean models that can predict systems level function for both linear and cyclic systems.

### The bipartite Boolean logic scales to large-scale systems

Finally, we applied the method on the pheromone response pathway of baker’s yeast. We chose this pathway to benchmark the bBM method due to the existence of an excellently annotated, comprehensive and mechanistically detailed rule based model (RBM).^20^ The original RBM contains 229 rules with 200 parameters (166 unknown) that define how 18 components can assume over 200.000 distinct states (http://yeastpheromonemodel.org/wiki/Extracting_the_model). While this is one of the most carefully built and curated RBMs, it remains difficult to meaningfully simulate it as such.^21^ Hence, it constitutes an excellent benchmark target for the bBM method.

**Fig. 6:**
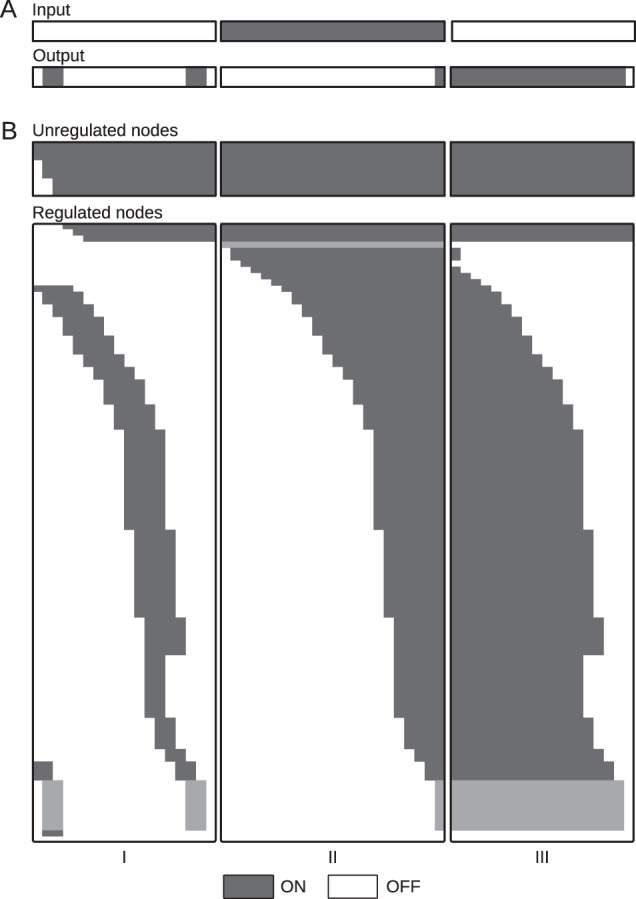
The updated pheromone model responds as expected to pheromone. We generated a bBM form the updated pheromone model and (I) simulated it in the absence of pheromone (unbound pheromone set to false) until the first steady state, where (II) free pheromone was set to true, representing pheromone stimulation, and the simulation repeated until next steady state was reached, before (III) pheromone was removed (by setting both free and bound pheromone to false) and the model simulated to the next steady state. The pathway turns on and off as expected, and finds a natural off state in the first simulation despite two activation pulses that go through the pathway. These are due to proteins that activate the pathway in their neutral states: the upstream Ste4, as discussed in the text, and the Ste12 transcription factor—which, according to the model, is active when not bound to Dig1 and Dig2. However, both pulses are transient and the steady state is robust against these transient dynamics. Panel (**a**) displays the input and output trajectories and panel (**b**) all the 248 state and reaction trajectories with the 149 unregulated targets condensed to three lines. Grey lines in (**b**) indicate the input/output states as displayed in (**a**).

We simulated the pheromone bBM using a standardised simulation workflow.^22^ The rxncon translation of the RBM, which is described elsewhere,^16^ is defined by 95 elemental reactions and 231 (non-zero) contingencies. We generated the bBM using the smoothed update rules, which produced a bipartite Boolean model with 130 reaction targets (due to separation of bidirectional reactions and duplication of degradation reactions) and 118 state targets. With 248 targets, the model is too large to use an exhaustive search of initial states (statespace = 2^248^; ca 10^74^ distinct configurations), so we fall back on our default initiation state vector (all neutral state targets are true, all generic component targets true, all other targets are false). From this initial state, we first simulated the model to a first point attractor, to let it find its natural “off state”, as explained for the HOG pathway above: thereafter, we iteratively switched the input to true and false. Analysing the outcome, we found that the pathway was constitutively active and unresponsive to pheromone. First, we examined if this was due to the interpretation of quantitative effects that are lost in the Boolean model. However, neither treating all quantitative (“K+”/“K−”) contingencies as absolute (“!”/“x”), nor treating them as no effect (“0”), solves the problem. Furthermore, the original RBM was never simulated and proven to be functional. Hence, we proceeded with the minimal model (treating quantitative contingencies as no effect) and looked deeper into the pathway behaviour, finding that it activates in the absence of signal due to constitutive release of Ste4, which represents the beta/gamma subunit of the trimeric G-protein at the top of the cascade, as well as constitutively free—and hence active—Ste12. To address these problems, we change four quantitative contingencies into qualitative contingencies. In addition, we needed to limit turnover of Ste4 bound Gpa1 (to prevent signal-independent release of unbound Ste4, the activator of the pathway) and to remove Fus3 dependent degradation of Ste12 which made the pathway “single-shot”. The final model with changes can be found in Supplementary Model 2, and the simulation trajectories are shown in Fig. 6. This updated version of the model responds to pheromone exposure and withdrawal as expected, despite containing 83 quantitative contingencies—which are ignored in the model generation—indicating that a much simpler model would suffice to capture the key features of the pathway.

## Discussion

Here, we present a qualitative simulation method for large-scale, mechanistically detailed signal transduction network models. The formalism is based on Boolean logic and can be simulated and studied by a standard package such as BoolNet. However, we present a fundamentally new concept to formulate Boolean models. First, we create a bipartite model at the level of elemental reactions and states, capturing the key elements in signal transduction at an appropriate resolution for mechanistic modelling of these processes. Second, based on detailed analysis of two minimal reaction motifs, and on a small set of standard assumptions, we define two generic update rules: one for reaction targets and one for state targets. These generic update rules map a bipartite rxncon network on a unique bBM with defined truth tables. The elemental reactions define the update rules for the state targets, and the contingencies define the update rules for the reaction targets. We show that these building blocks can be assembled like LEGO bricks into a bipartite Boolean model that predicts system-level function from molecular mechanisms, without optimisation at the system level.

The unique mapping from rxncon to an executable bBM that predicts system behaviour is highly non-trivial. Normally, it is relatively easy to build a Boolean model structure, but challenging to define truth tables that enable the model to reproduce the behaviour of the system.^23^ Here, we find that the regulatory structure encoded in the rxncon network already uniquely defines a Boolean model with set truth tables, and that this Boolean model meaningfully predicts system-level behaviour. Thus, the bBM logic we present here bridges the microscopic (biochemical reactions) and macroscopic (input-output) levels of cellular signal transduction, fulfilling the requirements for the cellular “mechanics” proposed by Hlavacek and Faeder—at least qualitatively.^6^

This has far-reaching implications: first, it provides an efficient validation tool in the model building process. This allows the model construction to be separated in to two phases: a qualitative and a quantitative phase. Boolean models are computationally inexpensive, and the automatic model generation supports iterative model creation, analysis and improvement. In addition, we are better equipped with knowledge at the qualitative level, suggesting that this level should be optimised first. As rxncon supports compilation into both RBMs and bBMs (as well as several graphical formats), it can be used to facilitate this process:^22^ the structural model can be created and validated using graphical tools and bBM simulation, and later the improved network can be used to create a rule-based model. Hence, the more expensive parameterisation cycles can be performed after the qualitative model has passed the validation process. Second, the bBM method can be used for validation of large-scale signal transduction networks. Previously, large-scale reconstruction of signal transduction has been limited to graphical maps that cannot be executed.^24–26^ The method we present here changes this: we can now validate—through simulation—large-scale reconstructions of signal transduction networks.

The bBM formalism we presented here is fundamentally different from its previous incarnation.^15^ First, it has been designed to capture system-level behaviour, not the states of individual molecule instances in the network, meaning that states that would be mutually exclusive on a single molecule can be true at the same time. As we show in Fig. 5, this is critical for meaningful system-level predictions. Second, we used a constructive approach, defining all possible behaviours at the level of two families of minimal reaction motifs, to design two generic update rules. These update rules can be used to map any rxncon system, even extended by new reaction types, through the interpretation of the flexible skeleton rule definition as synthesis, degradation, production or consumption of different states. Third, the method we present here inherits the expressiveness and flexibility of rxncon 2.0, including the explicit representation of neutral states.^16^ Fourth, the reimplementation has improved the model generation, enabled the use of different export options, and improved the model creation and analysis workflow. While the previous incarnation worked well in many instances,^15,27,28^ these models had issues with certain reaction types (most notably degradation) and spurious oscillations. That the latter appeared so rarely was due to the implicit dominance of modified states: neutral states were not explicitly represented, making modification or binding reactions dominant over reactions that returned components to their neutral state. Here, we eliminate this artificial hierarchy, which we consider undesirable, and explicitly include the neutral states. However, most of these states are constantly true in our simulations. This may have two reasons: first, it could reflect biology: there would be a constant pool of unmodified components as long as there is a constant turnover (and hence synthesis, which per definition occurs in the neutral states). Second, it could reflect an experimental bias, as we know much more about the modifying reactions than about the reactions that reverse the modification (e.g. phosphorylation vs dephosphorylation,^29^). If so, the formalism we present here helps us make this information bias explicit, and will allow us to integrate the regulation on these reactions as the knowledge becomes available.

Finally, the method enables a scale-shift in signal transduction modelling. Hitherto, executable signalling models have been mechanistically detailed or large-scale, but not both. Most mechanistic large-scale reconstructions are technically microstate models that could be simulated after parametrisation. However, they are actually divided into several unconnected modules and could hence not be simulated at the system level.^24–26^ Rule-based modelling languages have been used to build relatively large models,^21,30^ but even these models are limited to few (18 in these cases) components and parametrisation is already an outstanding challenge. In contrast, comprehensive signalling models will need to account for hundreds or thousands of signalling components, carrying many thousands of distinct elemental states. Here, we present a method that can deal with mechanistic signalling networks at this scope, and we have successfully used this method to build and analyse a comprehensive mechanistic model of the yeast cell division cycle, which accounts for 229 proteins, 790 elemental reactions and 1238 elemental states down to residue resolution when applicable^17^—far beyond the potential of previous mechanistic modelling. The method is qualitative, but this may be an advantage given the sparsity of reliable quantitative information on rate constants. In addition, even metabolic modelling—clearly the state of the art in genome-scale modelling—is limited to qualitative or semi-quantitative simulation methods at the genome scale.^31^

Taken together, we present a parameter-free model creation and simulation method for models of signal transduction. Unique models are generated directly from empirical data formalised in the rxncon 2.0 language, without need for fitting or optimisation. For the first time, we can simulate mechanistic models of signal transduction network at the genome scale.

## Methods

### rxncon installation and execution

The rxncon framework requires Python 3.5 or 3.6. Make sure you have one of these Python versions installed. Anaconda (https://www.continuum.io/downloads) provides an easy way to install the most current Python version. With Python installed and up to date, you are ready to install rxncon:

Under Windows:

1. Open the console and type “pip install rxncon”.^1^ The default installation folder will depend on your Python installation. With Anaconda, the rxncon folder appears in [user]/Anaconda3/lib/Site-packages. The files you will need to call appear in [user]/Anaconda3/Scripts.
2. To test the installation, navigate the console to the folder with the scripts and type “python rxncon2bngl.py”.^2^ Expect a string “Usage: rxncon2bngl.py [OPTIONS] EXCEL_FILE” and an error message “Error: Missing argument “excel_file”.

Under OS X:

1. Open the console and type ”pip install rxncon”. The default installation folder will depend on your Python installation. With Anaconda, the rxncon folder appears in [user]/Anaconda3/lib/python3.6/Site-packages. The files you will need to call appear in [user]/Anaconda3/bin.
2. To test the installation, navigate the console to the folder with the scripts and type “python rxncon2bngl.py”.^3^ Expect a string “Usage: rxncon2bngl.py [OPTIONS] EXCEL_FILE” and an error message “Error: Missing argument “excel_file”.

Under Linux:

1. Make sure you have PIP installed. If not, use your package manager to install it. E.g., on debian-based systems type “sudo apt install python3-pip”.
2. Open a terminal and type “pip3 install rxncon –user”. This installs into $HOME/.local, the executables are in $HOME/.local/bin.

3. To get easy access to the rxncon scripts, you can update your PATH environment variable to include this directory: put something like “export PATH=$HOME/.local/bin:$PATH” into your .bashrc.
4. To test the installation, type “rxncon2bngl.py”.^4^ Expect a string “Usage: rxncon2bngl.py [OPTIONS] EXCEL_FILE” and an error message “Error: Missing argument “excel_file”.

#### Boolnet installation

The logical simulation of rxncon networks uses BoolNet, an R package. To use these tools:

1. (Optional) Download and install R-studio (https://www.rstudio.com).^5^
2. Make sure you have R installed. R can be installed through Anaconda, by opening the console and typing: “conda install –c r r-essentials”.^6^
3. The BoolNet package can be installed from R. In the console, type “R” to enter the R environment. Then type “install.packages(“BoolNet”)” and select the download server.

#### Model creation and analysis

The creation of the rxncon models are described elsewhere. The High Osmolarity Glycerol (HOG) model was taken from^15^ and adapted to rxncon 2.0. The pheromone (PHER) model was translated from the yeastpheromonemodel.org wiki as described in.^16^ The bipartite Boolean model files were created with the rxncon compiler software, by calling the “rxncon2boolnet.py” script on the model (.xls) files with default setting:

python rxncon2boolnet.py path/model.xls

from the folder where the rxncon3.boolnet.py script is located, and where path/model.xls is the path to and file name of the rxncon model, with the file extension.

To access the possible options, call the script without a but with the –help command:

python rxncon2boolnet.py –help

This lists the possible options that can be appended to the call command, e.g:

python rxncon2boolnet.py path/model.xls –k_plus strict –k_minus strict

to run the model generation considering with quantitative contingencies (“K+”/“K−”) considered absolute (“!”/“x”) instead of being ignored.

The rxncon2boolnet.py script generates three files. First, the bipartite Boolean model file (<ModelName>.boolnet) contains the update rules using states and reaction IDs. Second, the symbol mapping file (<ModelName>_symbols.csv) defines which IDs correspond to which states and reactions in the rxncon file. Third, the initial vector (<ModelName>_initial_vals.csv’) sets the initial state of the Boolean simulation.

Model simulation was done with the R CRAN package BoolNet.^18^ To facilitate simulation, we prepared an R script that can be downloaded from https://github.com/rxncon/tools (BoolNetSim.R). To use this script through R studio:

- Save the network files and the R script into a single directory.
- Start RStudio.
- Open a new project and create it in the directory where you saved your files.
- Make sure your model files are located in the project folder.
- Open the R script. Set the filePrefix in the R script to <model>.
- Execute the entire script by selecting all text (ctrl+a) and pressing ctrl+enter.

The script generates five files: (i) <ModelName>.pdf, which graphically displays the simulation trajectory from initial state to the attractor, (ii) <ModelName>_trajectory_first.csv, with the trajectory as values (0/1) in tabular format, (iii) <ModelName>_2.pdf, which graphically displays the simulation trajectory from the attractor (useful to distinguish a point attractor (two columns) from a cyclic attractor (>2 columns), (iv) <ModelName>_trajectory_second.csv, with the second trajectory in tabular format, and (v) <ModelName>_new_attractor.csv, with the new attractor as an initial values file.

Where “<ModelName>” is the file name (without extension) of your rxncon model.

Within a Boolean model, we expect the output to be responsive to the input. The sign of the dependence does not matter, and we can start with the input either on or off. We simulate the model until it reaches an attractor. If it is a point attractor, we use it as starting point for the next simulation but turn the Input signal into a truth value, activating the output target and simulate again until we reach another attractor. We iteratively change inputs and simulate to an attractor state until we reach an attractor we have already seen. For a detailed discussion of the workflow, see^22^ (pre-print at arXiv: https://arxiv.org/abs/1802.01328).

#### Software and model availability

The rxncon software is open source, distributed under the lGPL licence, and can be installed from the python package index with “pip install rxncon” (code also available at https://github.com/rxncon/rxncon but without dependencies). The rxncon model files are available as Supplementary Model 1 (HOG model), Supplementary Model 2 (final pheromone model) or through download from https://github.com/rxncon/models/ (YeastPheromoneModel.xls (the initial pheromone model).

## Supplementary information

Supplementary Information

Supplementary Model 2

reporting-summary

## Data Availability

The models and code are freely available through the paper or public repositories. The rxncon software is open source, distributed under the lGPL licence, and can be installed from the python package index with “pip install rxncon”. The code is also available downloaded from https://github.com/rxncon/rxncon. The rxncon model files are available as Supplementary Model 1 (HOG model), Supplementary Model 2 (final pheromone model), Supplementary Model 3 (covalent motif), Supplementary Model 4 (interaction motif) or through download from https://github.com/rxncon/models/ (YeastPheromoneModel.xls (initial pheromone model)).

## Footnotes

1 On some computers, the installation of Pyeda fails for unknown reasons. In this case, try typing “pip install—no-cache-dir rxncon”.

2 In this case, by typing”cd Anaconda3/Scripts”.

3 In this case, by typing”cd Anaconda3/bin”.

4 This only works with the path set. Without this, type “=$HOME/.local/bin/rxncon2bngl.py”.

5 Depending on installation method, this may or may not come with R. If not, the location of R must be set in the dialogue box. If R is installed from Anaconda, the windows path would be “[user]/Anaconda3/R”.

6 If the conda command is not recognised, try to close and reopen the console.
