# Supplementary material for: A scalable method for parameter-free simulation and validation of mechanistic cellular signal transduction network models
Source: NPJ Syst Biol Appl. 2020 Jan 10;6:2. doi: 10.1038/s41540-019-0120-5 (PMC6954118; doi:10.1038/s41540-019-0120-5)
Supplement: Supplementary file 1 — Supplementary Information [file 41540_2019_120_MOESM1_ESM.pdf]

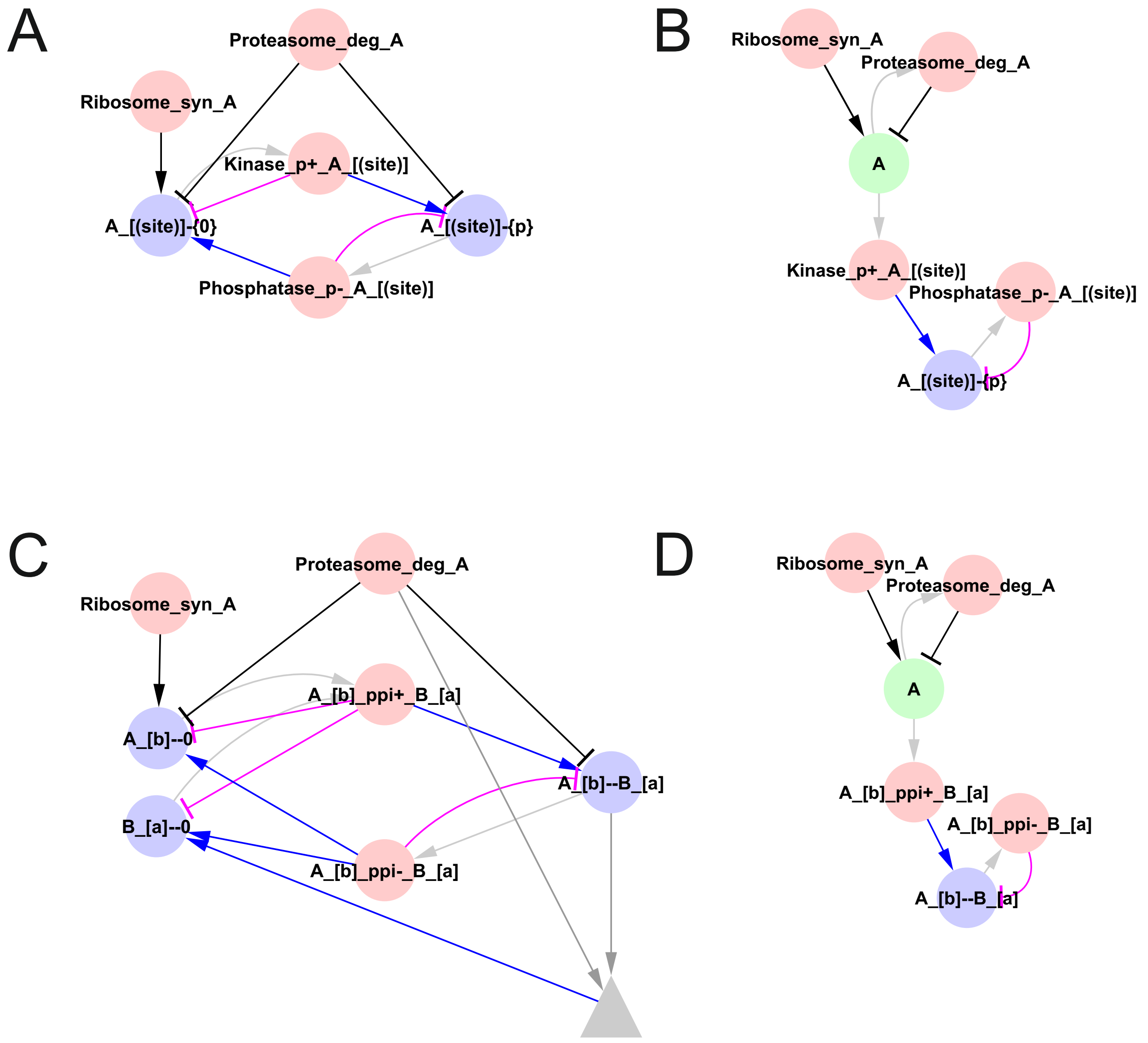

Supplementary Figure 1: The minimal reaction motifs as elemental species-reaction graphs and regulatory graphs. The minimal motifs from Figures 2 (A, B) and 3 (C, D) visualised as elemental species-reaction graphs (A, C) and regulatory graphs (B, D). Both graph formats visualise all four elemental reaction types: Synthesis (syn) and degradation (deg) of A, and either phosphorylation (P+) and dephosphorylation (P-) or binding to (ppi+) or dissociation from (ppi-) B, in the covalent modification and interaction motifs, respectively. The elemental species-reaction graph also include all elemental states: A with an empty ("A_[(site)]-{0}") or phosphorylated ("A_[(site)]-{P}) "site" residue in the covalent motif (panel A), or A and B unbound ("A_[b]--0"/"B_[a]--0") or the AB dimer ("A_[b]--B_[a]") (panel B). The grey triangle in (B) is an "AND" node, indicating that the reaction "Proteasome_deg_A" produces the B unbound to A ("B_[a]--0") when the AB dimer is degraded. The regulatory graphs (B, D) display the same motifs but excludes the neutral states. This makes the graphs much easier to read, especially for complex networks, but necessitates the inclusion of component nodes ("A") when components are turned over to indicate the dependence on a neutral state. The components only appear in the model if a component has no modification or interaction states; all other components are described by their actual states. Nodes visualise elemental reactions (red), elemental states (blue) or components (green). Edges visualise the effect of reactions on states (synthesis: black w. arrowhead, degradation: black w. "T", production: blue w. arrowhead, consumption: purple w. "T") and the effect of states on reactions (source state: grey w. arrowhead, contingencies (green for activation (K+/!) and red for inhibition (K-/x), not used in this example). Incoming edges to Boolean gates display complex requirements. Here, B (B_[a]--0) is released when the A component in the AB dimer is degraded.

Supplementary Discussion

This section contains an annotated version of the state target updates for the minimal modification motif (Figure 2), and a detailed illustration of the equations from the results section using the HOG pathway model as an example.

Exempli Gratia #1: Minimal modification motif state updates.

The minimal modification motif has four reactions and no contingencies:

!UID:Reaction	!ComponentA:Name	…	…	!Reaction	!ComponentB:Name	…	!ComponentB:Residue
Kinase_P+_A_[(site)]	Kinase			P+	A		site
Phosphatase_P-_A_[(site)]	Phosphatase			P-	A		site
Ribosome_syn_A	Ribosome			syn	A		
Proteasome_deg_A	Proteasome			deg	A		

The model generation (without smoothing) creates the following .boolnet file:

```
R0, (S2 & (S1 | S0))
R1, (S3 & (S1 | S0))
R2, (S4 & (S1 | S0))
R3, S5
S0, (R3 | (!(R2) & ((S0 & !((!(R2) & S0 & R0))) | (!(R2) & S1 & R1))
    & (S1 | S0)))
S1, ((!(R2) & ((S1 & !((!(R2) & S1 & R1))) | (R0 & !(R2) & S0)) &
    (S1 | S0)) | (R0 & R3))
S2, S2
S3, S3
S4, S4
S5, S5
```

The meaning of R0-R3 and S0-S5 can be found in the _symbols.csv file:

```
R0, Kinase_p+_A_[(site)]
R1, Phosphatase_p-_A_[(site)]
R2, Proteasome_deg_A
R3, Ribosome_syn_A
S0, A_[(site)]-{0}
S1, A_[(site)]-{p}
S2, Kinase
S3, Phosphatase
S4, Proteasome
S5, Ribosome
```

The interesting states are S0 (A unphosphorylated, referred to as $S_{A\text{-}\{0\}}$ below) and S1 (A phosphorylated, referred to as $S_{A\text{-}\{P\}}$ below), which have complex update rules. These are the states whose behaviour we analyse in figure 2, and below we explain these update rules in detail.

States: $S_{A\text{-}\{0\}}$ = unphosphorylated, $S_{A\text{-}\{P\}}$ = phosphorylated

Reactions: R_{syn} = synthesis (synthesises A-{0}), R_{deg} = degradation (degrades A-{0} and A-{P}), R_{p+} = phosphorylation (produces A-{P}, consumes A-{0}), R_{p-} = dephosphorylation (produces A-{0}, consumes A-{P})

The neutral state $S_{A\text{-}\{0\}\ldots}$:

$S_{A\text{-}\{0\}}$ = R_{SYN} OR(	… is true if **synthesised** OR if
NOT R_{DEG} AND	not **degraded** AND
($S_{A\text{-}\{0\}}$ OR $S_{A\text{-}\{P\}}$) AND (	the component is present and
(	either
R_{P-} AND $S_{A\text{-}\{P\}}$ AND	**produced through dephosphorylation** of
NOT R_{DEG}	$S_{A\text{-}\{P\}}$ in the absence of degradation*
) OR	or
(	
$S_{A\text{-}\{0\}}$ AND NOT	it **is present**
(R_{P+} AND $S_{A\text{-}\{0\}}$ AND	**and not consumed** by phosphorylation of
NOT R_{DEG})	$S_{A\text{-}\{0\}}$ in the absence of degradation*
)	
)	
)	

The above has been reordered and edited (to remove unnecessary brackets) for readability. It aligns to the automatically generated expression as follows:

```
Line 1:    S0, (R3 |
Line 2:    (!(R2) &
Line 9:    ((S0 & !
Line 11:   ((!(R2)
Line 10:   & S0 & R0))) |
Line 6:   (!(R2)
Line 5:  & S1 & R1))
Line 3:  & (S1 | S0)))
```

Similarly, the modification state $S_{A\text{-}\{P\}...}$:

$S_{A\text{-}\{P\}}$ = (R_{SYN} AND R_{P+}) OR (	… is true if **indirectly synthesised** OR if
NOT R_{DEG} AND	not **degraded** AND
($S_{A\text{-}\{0\}}$ | $S_{A\text{-}\{P\}}$) AND (	the component is present and
(	either
R_{P+} AND $S_{A\text{-}\{0\}}$ AND	**produced through phosphorylation** of
NOT R_{DEG}	$S_{A\text{-}\{0\}}$ in the absence of degradation*
) OR	or
(	
$S_{A\text{-}\{P\}}$ AND NOT	it **is present**
(R_{P-} AND $S_{A\text{-}\{P\}}$ AND	**and not consumed** by dephosphorylation
NOT R_{DEG})	of $S_{A\text{-}\{P\}}$ in the absence of degradation*
)	
)	
)	

Note that the degradation checks in the production/consumption terms (marked with *) are not redundant, although it appears so here. They check for source state degradation - these are necessary if the source state belong on another component (e.g. in binding reactions) or if certain states are protected from degradation. However, in this simple example they appear (and are) redundant.

The remaining four states (S2-S5) correspond to the catalysts of the four reactions and are constantly true.

The reaction updates are straightforward as there are no contingencies: They only require the reacting components to be true: I.e., the catalysts and, except in the case of synthesis, component A - which is present if either the phosphorylated or unphosphorylated state is true.

However, we do not use these reaction update rules in the test: We replace the reaction updates with a constant true/false value in the model to generate 16 (2^4) permutations, which, in combination with the four permutations of initial values for $S_{A\text{-}\{0\}}$ and $S_{A\text{-}\{P\}}$, generates 64 model variants.

The initial values are changed in the _initial_vals.csv file, which by default reads:

```
R0, 0      , #  Kinase_p+_A_[(site)]
R1, 0      , #  Phosphatase_p-_A_[(site)]
```

```
R2, 0      , #  Proteasome_deg_A
R3, 0      , #  Ribosome_syn_A
S0, 1      , #  A_[(site)]-{0}
S1, 0      , #  A_[(site)]-{p}
S2, 1      , #  Kinase
S3, 1      , #  Phosphatase
S4, 1      , #  Proteasome
S5, 1      , #  Ribosome
```

The interaction motif analysis (Figure 3) is performed the same way, except that we get 128 permutations as there are four reactions and three elemental states involved. The minimal motif models are attached as Supplementary Models 3 (covalent modification motif) and 4 (interaction motif).

Smoothed expressions

With smoothing, the model generation creates the following .boolnet file:

```
R0, (S2 & (S0 | S1))
R1, (S3 & (S0 | S1))
R2, (S4 & (S0 | S1))
R3, S5
S0, (R3 | (!(R2) & ((S0 & !((!(R2) & S0 & R0))) | (R1 & ((!(R2) &
    ((S1 & !((!(R2) & S1 & R1))) | (!(R2) & S0 & R0)) & (S0 | S1)) |
    (R3 & R0) | (!(R2) & S1)))) & (S0 | S1)))
S1, ((!(R2) & ((S1 & !((!(R2) & S1 & R1))) | (R0 & (R3 | (!(R2) &
    ((S0 & !((R0 & !(R2) & S0))) | (!(R2) & S1 & R1)) & (S0 | S1)) |
    (!(R2) & S0)))) & (S0 | S1)) | (R0 & R3))
S2, S2
S3, S3
S4, S4
S5, S5
```

The only difference is in the S0/S1 lines, where the smoothing terms appear in the source state expression. Compare the smoothed (lower) with the non-smoothed (upper) expression.

```
S0, (R3 | (!(R2) & ((S0 & !((!(R2) & S0 & R0))) | (!(R2) & S1 & R1))
    & (S1 | S0)))
S1, ((!(R2) & ((S1 & !((!(R2) & S1 & R1))) | (R0 & !(R2) & S0)) &
    (S1 | S0)) | (R0 & R3))

S0, (R3 | (!(R2) & ((S0 & !((!(R2) & S0 & R0))) | (R1 & ((!(R2) &
    ((S1 & !((!(R2) & S1 & R1))) | (!(R2) & S0 & R0)) & (S0 | S1)) |
    (R3 & R0) | (!(R2) & S1)))) & (S0 | S1)))
S1, ((!(R2) & ((S1 & !((!(R2) & S1 & R1))) | (R0 & (R3 | (!(R2) &
    ((S0 & !((R0 & !(R2) & S0))) | (!(R2) & S1 & R1)) & (S0 | S1)) |
    (!(R2) & S0)))) & (S0 | S1)) | (R0 & R3))
```

For S0, dephosphorylation (R1) now triggers under the following conditions: The reaction (R1) is true and the source state (S1) is (1) indirectly synthesised, (2) produced by phosphorylation in the absence of degradation, or (3) present and not consumed or degraded. 3b is the original term from the unsmoothed export and is redundant with the smoothed expression.

```
R1 & (
      (!(R2) & (
           (S1 & !((!(R2) & S1 & R1))) |    (3a)
           (!(R2) & S0 & R0)                (2)
      ) & (S0 | S1)) |
      (R3 & R0) |                           (1)
      (!(R2) & S1)                          (3b)
     )
```

For S1, phosphorylation (R0) now triggers under the following conditions: The reaction (R0) is true and the source state (S0) is (1) indirectly synthesised, (2) produced by dephosphorylation in the absence of degradation, or (3) present and not consumed or degraded. 3b is the original term from the unsmoothed export and is redundant with the smoothed expression.

```
R0 & (
      R3 |                                 (1)
      (!(R2) &
        ((S0 & !((R0 & !(R2) & S0))) |     (3a)
        (!(R2) & S1 & R1)) &               (2)
      (S0 | S1)) |
      (!(R2) & S0))                        (3b)
```

Exempli Gratia #2: The Hog pathway model generation.

In the following, we exemplify the application of the equations on the Hog pathway model. For improved readability, components, reactions, states and inputs are prefixed with C, R, S and I, with their actual names written in subscript.

Eq.1: Components $C_i = \bigcap_{\substack{site\ j\ on \\ component\ i}} \bigcup_{\substack{state\ k\ on \\ site\ j}} S_k$

Most components (in this particular model) have a single site (residue or domain) that can be in the neutral or modified/bound state. Their component expressions consist of a simple OR statement between the neutral and modified/bound states:

C_{Sln1} = $S_{Sln1\_[(Site)]\text{-}\{0\}}$ **OR** $S_{Sln1\_[(Site)]\text{-}\{p\}}$

C_{Ypd1} = $S_{Ypd1\_[(H64)]\text{-}\{0\}}$ **OR** $S_{Ypd1\_[(H64)]\text{-}\{p\}}$

C_{Ssk2} = $S_{Ssk2\_[ssk1]\text{--}0}$ **OR** $S_{Ssk1\_[RR]\text{--}Ssk2\_[ssk1]}$

C_{Pbs2} = $S_{Pbs2\_[(Ssk2Site)]\text{-}\{0\}}$ **OR** $S_{Pbs2\_[(Ssk2Site)]\text{-}\{p\}}$

C_{Hog1} = $S_{Hog1\_[(Pbs2Site)]\text{-}\{0\}}$ **OR** $S_{Hog1\_[(Pbs2Site)]\text{-}\{p\}}$

C_{Hot1} = $S_{Hot1\_[(Hog1Site)]\text{-}\{0\}}$ **OR** $S_{Hot1\_[(Hog1Site)]\text{-}\{p\}}$

One of the components has two distinct sites in the model; one residue and one domain. The component expression is then written as an AND of two OR statements.

C_{Ssk1} = ($S_{Ssk1\_[(D544)]\text{-}\{0\}}$ **OR** $S_{Ssk1\_[(D544)]\text{-}\{p\}}$) **AND** ($S_{Ssk1\_[RR]\text{--}0}$ **OR** $S_{Ssk1\_[RR]\text{--}Ssk2\_[ssk1]}$)

This means that at a protein is considered degraded as soon as all states for a single site have disappeared.

Finally, there is one component (PPT) in the system that does not carry any states. This is included in the model with a component state:

C_{PPT}

Eq. 2. Components in each reaction: $K(R_i) = \bigcap_{\substack{component\ j \\ reacts\ in\ R_i}} C_j$

The first reaction is mono-molecular, hence only one component is necessary:

$K(R_{Sln1\_ap+\_Sln1\_[(Site)]})$ = C_{Sln1}

The remaining reactions are bi-molecular, containing two components each:

$K(R_{Sln1\_[(Site)]\_pt\_Ypd1\_[(H64)]}) = C_{Sln1}$ AND C_{Ypd1}

$K(R_{Ypd1\_[(H64)]\_pt\_Ssk1\_[(D544)]}) = C_{Ypd1}$ AND C_{Ssk1}

$K(R_{Ssk1\_[RR]\_ppi+\_Ssk2\_[ssk1]}) = C_{Ssk1}$ AND C_{Ssk2}

$K(R_{Ssk1\_[RR]\_ppi-\_Ssk2\_[ssk1]}) = C_{Ssk1}$ AND C_{Ssk2}

$K(R_{Ssk2\_p+\_Pbs2\_[(Ssk2Site)]}) = C_{Ssk2}$ AND C_{Pbs2}

$K(R_{Pbs2\_p+\_Hog1\_[(Pbs2Site)]}) = C_{Pbs2}$ **AND** C_{Hog1}

$K(R_{Hog1\_p+\_Hot1\_[(Hog1Site)]}) = C_{Hog1}$ **AND** C_{Hot1}

$K(R_{PPT\_p-\_Ssk1\_[(D544)]}) = C_{PPT}$ **AND** C_{Ssk1}

$K(R_{PPT\_p-\_Pbs2\_[(Ssk2Site)]}) = C_{PPT}$ **AND** C_{Pbs2}

$K(R_{PPT\_p-\_Hog1\_[(Pbs2Site)]}) = C_{PPT}$ **AND** C_{Hog1}

$K(R_{PPT\_p-\_Hot1\_[(Hog1Site)]}) = C_{PPT}$ **AND** C_{Hot1}

Eq. 3. Components in each state: $K(S_i) = \bigcap_{\substack{component\ j \\ carries\ S_i}} C_j$

All neutral states are carried by a single component, regardless of if the site is a domain or a residue:

$K(S_{Sln1\_[(Site)]-\{0\}}) = C_{Sln1}$

$K(S_{Ypd1\_[(H64)]-\{0\}}) = C_{Ypd1}$

$K(S_{Ssk1\_[(D544)]-\{0\}}) = C_{Ssk1}$

$K(S_{Ssk1\_[RR]--0}) = C_{Ssk1}$

$K(S_{Ssk2\_[ssk1]--0}) = C_{Ssk2}$

$K(S_{Pbs2\_[(Ssk2Site)]-\{0\}}) = C_{Pbs2}$

$K(S_{Hog1\_[(Pbs2Site)]-\{0\}}) = C_{Hog1}$

$K(S_{Hot1\_[(Hog1Site)]-\{0\}}) = C_{Hot1}$

All modification states are carried by a single component:

$K(S_{Sln1\_[(Site)]-\{p\}}) = C_{Sln1}$

$K(S_{Ypd1\_[(H64)]-\{p\}})= C_{Ypd1}$

$K(S_{Ssk1\_[(D544)]-\{p\}}) = C_{Ssk1}$

$K(S_{Pbs2\_[(Ssk2Site)]-\{p\}}) = C_{Pbs2}$

$K(S_{Hog1\_[(Pbs2Site)]-\{p\}}) = C_{Hog1}$

$K(S_{Hot1\_[(Hog1Site)]-\{p\}}) = C_{Hot1}$

All bond states are carried by two components:

$K(S_{Ssk1\_[RR]--Ssk2\_[ssk1]}) = C_{Ssk1}$ AND C_{Ssk2}

Eq. 4. Reactions and source state(s): $R'_i = R_i \cap_{S_j \, consumed \, by \, R_i} S_j$

Forward reactions (autophoshorylation (AP+), phosphorylation (P+), protein-protein interaction (ppi+)) consume neutral states, reverse reactions (dephosphorylation (P-), protein-protein dissociation (ppi-)) consume modified/bound states. The phosphotransfer reactions (PT, line 2 and 3) are special as they consume one modified and one neutral state.

$R'_{Sln1\_ap+\_Sln1\_[(Site)]} = R_{Sln1\_ap+\_Sln1\_[(Site)]}$ AND $S_{Sln1\_[(Site)]-\{0\}}$

$R'_{Sln1\_[(Site)]\_pt\_Ypd1\_[(H64)]} = R_{Sln1\_[(Site)]\_pt\_Ypd1\_[(H64)]}$ AND ($S_{Sln1\_[(Site)]-\{p\}}$ AND $S_{Ypd1\_[(H64)]-\{0\}}$)

$R'_{Ypd1\_[(H64)]\_pt\_Ssk1\_[(D544)]} = R_{Ypd1\_[(H64)]\_pt\_Ssk1\_[(D544)]}$ AND($S_{Ypd1\_[(H64)]-\{p\}}$ AND $S_{Ssk1\_[(D544)]-\{0\}}$)

$R'_{Ssk1\_[RR]\_ppi+\_Ssk2\_[ssk1]} = R_{Ssk1\_[RR]\_ppi+\_Ssk2\_[ssk1]}$ AND ($S_{Ssk1\_[RR]--0}$ AND $S_{Ssk2\_[ssk1]--0}$)

$R'_{Ssk1\_[RR]\_ppi-\_Ssk2\_[ssk1]} = R_{Ssk1\_[RR]\_ppi-\_Ssk2\_[ssk1]}$ AND $S_{Ssk1\_[RR]--Ssk2\_[ssk1]}$

$R'_{Ssk2\_p+\_Pbs2\_[(Ssk2Site)]} = R_{Ssk2\_p+\_Pbs2\_[(Ssk2Site)]}$ AND $S_{Pbs2\_[(Ssk2Site)]-\{0\}}$

$R'_{Pbs2\_p+\_Hog1\_[(Pbs2Site)]} = R_{Pbs2\_p+\_Hog1\_[(Pbs2Site)]}$ AND $S_{Hog1\_[(Pbs2Site)]-\{0}$

$R'_{Hog1\_p+\_Hot1\_[(Hog1Site)]} = R_{Hog1\_p+\_Hot1\_[(Hog1Site)]}$ AND $S_{Hot1\_[(Hog1Site)]-\{0\}}$

$R'_{PPT\_p-\_Ssk1\_[(D544)]} = R_{PPT\_p-\_Ssk1\_[(D544)]}$ AND $S_{Ssk1\_[(D544)]-\{p\}}$

$R'_{PPT\_p-\_Pbs2\_[(Ssk2Site)]} = R_{PPT\_p-\_Pbs2\_[(Ssk2Site)]}$ AND $S_{Pbs2\_[(Ssk2Site)]-\{p\}}$

$R'_{PPT\_p-\_Hog1\_[(Pbs2Site)]} = R_{PPT\_p-\_Hog1\_[(Pbs2Site)}$ AND $S_{Hog1\_[(Pbs2Site)]-\{p\}}$

$R'_{PPT\_p-\_Hot1\_[(Hog1Site)]} = R_{PPT\_p-\_Hot1\_[(Hog1Site)]}$ AND $S_{Hot1\_[(Hog1Site)]-\{p\}}$

Note that the number of reactions is higher in the Boolean model (12) than in the rxncon table (11) due to the split of the bidirectional protein-protein interaction (ppi) in forward (ppi+) and a reverse (ppi-) reaction in the Boolean model.

Reactions may be true even in the absence of their source states, but they can only fire if all their source states are available.

Eq. 5. Synthesis: $$\Sigma(S_i) = \begin{cases} \bigcup_{S_i} R_{j\ synthesizes\ R'_j} & \text{for neutral states } S_i \\ \bigcup_{N(S_i)} R_{j\ synthesizes\ R'_j} \cap \bigcup_{S_i} R_{k\ produces\ R'_k} & \text{for non-neutral states } S_i \end{cases}$$

The Hog pathway model does not include synthesis or degradation of components.

Eq. 6. Contingencies: $$L(R_i) = \bigcap_j L_j^!(R_i) \bigcap_k \overline{L_k^{\times}(R_i)}$$

There are five regulated reactions in the Hog pathway model. The first depend on the model input (Turgor), and the four other on the state of one of the reactants.

$L(R_{Sln1\_ap+\_Sln1\_[(Site)]}) = I_{Turgor}$

$L(R_{Ssk1\_[RR]\_ppi+\_Ssk2\_[ssk1]}) = \text{NOT } S_{Ssk1\_[(D544)]-\{p\}}$

$L(R_{Ssk2\_p+\_Pbs2\_[(Ssk2Site)]}) = S_{Ssk1\_[RR]--Ssk2\_[ssk1]}$

$L(R_{Pbs2\_p+\_Hog1\_[(Pbs2Site)]}) = S_{Pbs2\_[(Ssk2Site)]-\{p\}}$

$L(R_{Hog1\_p+\_Hot1\_[(Hog1Site)]}) = S_{Hog1\_[(Pbs2Site)]-\{p\}}$

All other reactions in the model are regulated on the level of substrate availability. I.e., they are always true (as long as the components are initiated as true, as there is no synthesis or degradation of components) and fire as soon as all the source states are available.

Eq. 7. Reaction updates: $$R_i(t+1) = K(R_i;t) \cap L(R_i;t)$$

Reactions are active when the reacting component(s) are present **and** all absolute contingencies are fulfilled.

$R_{Sln1\_ap+\_Sln1\_[(Site)]} = C_{Sln1} \text{ AND } I_{Turgor}$

$R_{Sln1\_[(Site)]\_pt\_Ypd1\_[(H64)]} = C_{Sln1} \text{ AND } C_{Ypd1}$

$R_{Ypd1\_[(H64)]\_pt\_Ssk1\_[(D544)]} = C_{Ssk1} \text{ AND } C_{Ypd1}$

$R_{Ssk1\_[RR]\_ppi+\_Ssk2\_[ssk1]} = (C_{Ssk1} \text{ AND } C_{Ssk2}) \text{ AND NOT } S_{Ssk1\_[(D544)]-\{p\}}$

$R_{Ssk1\_[RR]\_ppi-\_Ssk2\_[ssk1]} = C_{Ssk1} \text{ AND } C_{Ssk2}$

$R_{Ssk2\_p+\_Pbs2\_[(Ssk2Site)]} = (C_{Ssk2} \text{ AND } C_{Pbs2}) \text{ AND } S_{Ssk1\_[RR]--Ssk2\_[ssk1]}$

$R_{Pbs2\_p+\_Hog1\_[(Pbs2Site)]} = (C_{Hog1} \textbf{ AND } C_{Pbs2}) \textbf{ AND } S_{Pbs2\_[(Ssk2Site)]-\{p\}}$

$R_{\text{Hog1\_p+\_Hot1\_[(Hog1Site)]}}$ = (C_{Hog1} **AND** C_{Hot1}) **AND** $S_{\text{Hog1\_[(Pbs2Site)]-\{p\}}}$

$R_{\text{PPT\_p-\_Ssk1\_[(D544)]}}$ = C_{PPT} **AND** C_{Ssk1}

$R_{\text{PPT\_p-\_Pbs2\_[(Ssk2Site)]}}$ = C_{PPT} **AND** C_{Pbs2}

$R_{\text{PPT\_p-\_Hog1\_[(Pbs2Site)]}}$ = C_{PPT} **AND** C_{Hog1}

$R_{\text{PPT\_p-\_Hot1\_[(Hog1Site)]}}$ = C_{PPT} **AND** C_{Hot1}

Eq. 8. State updates:

$$S_i(t+1) = \Sigma(t) \bigcup \left(K(S_i;t) \bigcap_{\substack{R_k degrades \\ S_i}} \overline{R'_k}(t) \bigcap \left\{ \bigcup_{\substack{R_l\, produces \\ S_i}} R'_l(t) \bigcup \left[S_i(t) \bigcap_{\substack{R_m consumes \\ S_i}} \overline{R'_m}(t) \right] \right\} \right)$$

As no synthesis or degradation reactions are considered in the Hog model, the components are constitutive and equation 8 simplifies to:

$$S_i(t+1) = K(S_i;t) \bigcap \left\{ \bigcup_{\substack{R_l\, produces \\ S_i}} R'_l(t) \bigcup \left[S_i(t) \bigcap_{\substack{R_m consumes \\ S_i}} \overline{R'_m}(t) \right] \right\}$$

I.e., all components in the state must be true and the state must either be produced (a producing reaction must be true AND the source state for that reaction must be true) OR the state must be present and not consumed (the state is true AND either the consuming reaction OR one of its source states must be false).

$S_{\text{Sln1\_[(Site)]-\{0\}}}$ = C_{Sln1} AND ($R'_{\text{Sln1\_[(Site)]\_pt\_Ypd1\_[(H64)]}}$ OR ($S_{\text{Sln1\_[(Site)]-\{0\}}}$ AND NOT $R'_{\text{Sln1\_ap+\_Sln1\_[(Site)]}}$))

$S_{\text{Ypd1\_[(H64)]-\{0\}}}$ = C_{Ypd1} AND ($R'_{\text{Ypd1\_[(H64)]\_pt\_Ssk1\_[(D544)]}}$ OR ($S_{\text{Ypd1\_[(H64)]-\{0\}}}$ AND NOT $R'_{\text{Sln1\_[(Site)]\_pt\_Ypd1\_[(H64)]}}$))

$S_{\text{Ssk1\_[(D544)]-\{0\}}}$ = C_{Ssk1} AND ($R'_{\text{PPT\_p-\_Ssk1\_[(D544)]}}$ OR ($S_{\text{Ssk1\_[(D544)]-\{0\}}}$ AND NOT $R'_{\text{Ypd1\_[(H64)]\_pt\_Ssk1\_[(D544)]}}$))

$S_{\text{Ssk1\_[RR]--0}}$ = C_{Ssk1} AND ($R'_{\text{Ssk1\_[RR]\_ppi-\_Ssk2\_[ssk1]}}$ OR ($S_{\text{Ssk1\_[RR]--0}}$ AND NOT $R'_{\text{Ssk1\_[RR]\_ppi+\_Ssk2\_[ssk1]}}$))

$S_{\text{Ssk2\_[ssk1]--0}}$ = C_{Ssk2} AND ($R'_{\text{Ssk1\_[RR]\_ppi-\_Ssk2\_[ssk1]}}$ OR ($S_{\text{Ssk2\_[ssk1]--0}}$ AND NOT $R'_{\text{Ssk1\_[RR]\_ppi+\_Ssk2\_[ssk1]}}$))

$S_{\text{Pbs2\_[(Ssk2Site)]-\{0\}}}$ = C_{Pbs2} AND ($R'_{\text{PPT\_p-\_Pbs2\_[(Ssk2Site)]}}$ OR ($S_{\text{Pbs2\_[(Ssk2Site)]-\{0\}}}$ AND NOT $R'_{\text{Ssk2\_p+\_Pbs2\_[(Ssk2Site)]}}$))

$S_{\text{Hog1\_[(Pbs2Site)]-\{0\}}}$ = C_{Hog1} AND ($R'_{\text{PPT\_p-\_Hog1\_[(Pbs2Site)]}}$ OR ($S_{\text{Hog1\_[(Pbs2Site)]-\{0\}}}$ AND NOT $R'_{\text{Pbs2\_p+\_Hog1\_[(Pbs2Site)]}}$))

$S_{\text{Hot1\_[(Hog1Site)]-\{0\}}}$ = C_{Hot1} AND ($R'_{\text{PPT\_p-\_Hot1\_[(Hog1Site)]}}$ OR ($S_{\text{Hot1\_[(Hog1Site)]-\{0\}}}$ AND NOT $R'_{\text{Hog1\_p+\_Hot1\_[(Hog1Site)]}}$))

$S_{\text{Sln1\_[(Site)]-\{p\}}}$ = C_{Sln1} AND ($R'_{\text{Sln1\_ap+\_Sln1\_[(Site)]}}$ OR ($S_{\text{Sln1\_[(Site)]-\{p\}}}$ AND NOT $R'_{\text{Sln1\_[(Site)]\_pt\_Ypd1\_[(H64)]}}$))

$S_{\text{Ypd1\_[(H64)]-\{p\}}}$ = C_{Ypd1} AND ($R'_{\text{Sln1\_[(Site)]\_pt\_Ypd1\_[(H64)]}}$ OR ($S_{\text{Ypd1\_[(H64)]-\{p\}}}$ AND NOT $R'_{\text{Ypd1\_[(H64)]\_pt\_Ssk1\_[(D544)]}}$))

$S_{\text{Ssk1\_[(D544)]-\{p\}}}$ = C_{Ssk1} AND ($R'_{\text{Ypd1\_[(H64)]\_pt\_Ssk1\_[(D544)]}}$ OR ($S_{\text{Ssk1\_[(D544)]-\{p\}}}$ AND NOT $R'_{\text{PPT\_p-\_Ssk1\_[(D544)]}}$))

$S_{\text{Pbs2\_[(Ssk2Site)]-\{p\}}}$ = C_{Pbs2} AND ($R'_{\text{Ssk2\_p+\_Pbs2\_[(Ssk2Site)]}}$ OR ($S_{\text{Pbs2\_[(Ssk2Site)]-\{p\}}}$ AND NOT $R'_{\text{PPT\_p-\_Pbs2\_[(Ssk2Site)]}}$))

$S_{\text{Hog1\_[(Pbs2Site)]-\{p\}}}$ = C_{Hog1} AND ($R'_{\text{Pbs2\_p+\_Hog1\_[(Pbs2Site)]}}$ OR ($S_{\text{Hog1\_[(Pbs2Site)]-\{p\}}}$ AND NOT $R'_{\text{PPT\_p-\_Hog1\_[(Pbs2Site)]}}$))

$S_{\text{Hot1\_[(Hog1Site)]-\{p\}}}$ = C_{Hot1} AND ($R'_{\text{Hog1\_p+\_Hot1\_[(Hog1Site)]}}$ OR ($S_{\text{Hot1\_[(Hog1Site)]-\{p\}}}$ AND NOT $R'_{\text{PPT\_p-\_Hot1\_[(Hog1Site)]}}$))

$S_{\text{Ssk1\_[RR]--Ssk2\_[ssk1]}}$ = (C_{Ssk1} AND C_{Ssk2}) AND ($R'_{\text{Ssk1\_[RR]\_ppi+\_Ssk2\_[ssk1]}}$ OR ($S_{\text{Ssk1\_[RR]--Ssk2\_[ssk1]}}$ AND NOT $R'_{\text{Ssk1\_[RR]\_ppi-\_Ssk2\_[ssk1]}}$))

We write these expressions out in detail for four examples, by substituting first for R'…:

$S_{\text{Sln1\_[(Site)]-\{0\}}}$ = C_{Sln1} AND (($R_{\text{Sln1\_[(Site)]\_pt\_Ypd1\_[(H64)]}}$ AND ($S_{\text{Sln1\_[(Site)]-\{p\}}}$ AND $S_{\text{Ypd1\_[(H64)]-\{0\}}}$)) OR ($S_{\text{Sln1\_[(Site)]-\{0\}}}$ AND NOT ($R_{\text{Sln1\_ap+\_Sln1\_[(Site)]}}$ AND $S_{\text{Sln1\_[(Site)]-\{0\}}}$)))

$S_{\text{Sln1\_[(Site)]-\{p\}}}$ = C_{Sln1} AND (($R_{\text{Sln1\_ap+\_Sln1\_[(Site)]}}$ AND $S_{\text{Sln1\_[(Site)]-\{0\}}}$) OR ($S_{\text{Sln1\_[(Site)]-\{p\}}}$ AND NOT ($R_{\text{Sln1\_[(Site)]\_pt\_Ypd1\_[(H64)]}}$ AND ($S_{\text{Sln1\_[(Site)]-\{p\}}}$ AND $S_{\text{Ypd1\_[(H64)]-\{0\}}}$))))

$S_{\text{Ssk1\_[RR]--0}}$ = C_{Ssk1} AND (($R_{\text{Ssk1\_[RR]\_ppi-\_Ssk2\_[ssk1]}}$ AND $S_{\text{Ssk1\_[RR]--Ssk2\_[ssk1]}}$) OR ($S_{\text{Ssk1\_[RR]--0}}$ AND NOT ($R_{\text{Ssk1\_[RR]\_ppi+\_Ssk2\_[ssk1]}}$ AND ($S_{\text{Ssk1\_[RR]--0}}$ AND $S_{\text{Ssk2\_[ssk1]--0}}$))))

$S_{\text{Ssk1\_[RR]--Ssk2\_[ssk1]}}$ = C_{Ssk1} AND C_{Ssk2} AND (($R_{\text{Ssk1\_[RR]\_ppi+\_Ssk2\_[ssk1]}}$ AND ($S_{\text{Ssk1\_[RR]--0}}$ AND $S_{\text{Ssk2\_[ssk1]--0}}$)) OR ($S_{\text{Ssk1\_[RR]--Ssk2\_[ssk1]}}$ AND NOT ($R_{\text{Ssk1\_[RR]\_ppi-\_Ssk2\_[ssk1]}}$ AND $S_{\text{Ssk1\_[RR]--Ssk2\_[ssk1]}}$)))

…and then for components, to arrive at an expression using only reaction and states:

$S_{\text{Sln1\_[(Site)]-\{0\}}}$ = ($S_{\text{Sln1\_[(Site)]-\{0\}}}$ **OR** $S_{\text{Sln1\_[(Site)]-\{p\}}}$) AND (($R_{\text{Sln1\_[(Site)]\_pt\_Ypd1\_[(H64)]}}$ AND ($S_{\text{Sln1\_[(Site)]-\{p\}}}$ AND $S_{\text{Ypd1\_[(H64)]-\{0\}}}$)) OR ($S_{\text{Sln1\_[(Site)]-\{0\}}}$ AND NOT ($R_{\text{Sln1\_ap+\_Sln1\_[(Site)]}}$ AND $S_{\text{Sln1\_[(Site)]-\{0\}}}$)))

$S_{\text{Sln1\_[(Site)]-\{p\}}}$ = ($S_{\text{Sln1\_[(Site)]-\{0\}}}$ **OR** $S_{\text{Sln1\_[(Site)]-\{p\}}}$) AND (($R_{\text{Sln1\_ap+\_Sln1\_[(Site)]}}$ AND $S_{\text{Sln1\_[(Site)]-\{0\}}}$) OR ($S_{\text{Sln1\_[(Site)]-\{p\}}}$ AND NOT ($R_{\text{Sln1\_[(Site)]\_pt\_Ypd1\_[(H64)]}}$ AND ($S_{\text{Sln1\_[(Site)]-\{p\}}}$ AND $S_{\text{Ypd1\_[(H64)]-\{0\}}}$))))

$S_{\text{Ssk1\_[RR]--0}}$ = (($S_{\text{Ssk1\_[(D544)]-\{0\}}}$ **OR** $S_{\text{Ssk1\_[(D544)]-\{p\}}}$) **AND** ($S_{\text{Ssk1\_[RR]--0}}$ **OR** $S_{\text{Ssk1\_[RR]--Ssk2\_[ssk1]}}$)) AND (($R_{\text{Ssk1\_[RR]\_ppi-\_Ssk2\_[ssk1]}}$ AND $S_{\text{Ssk1\_[RR]--Ssk2\_[ssk1]}}$) OR ($S_{\text{Ssk1\_[RR]--0}}$ AND NOT ($R_{\text{Ssk1\_[RR]\_ppi+\_Ssk2\_[ssk1]}}$ AND ($S_{\text{Ssk1\_[RR]--0}}$ AND $S_{\text{Ssk2\_[ssk1]--0}}$))))

$S_{\text{Ssk1\_[RR]--Ssk2\_[ssk1]}}$ = ((($S_{\text{Ssk1\_[(D544)]-\{0\}}}$ **OR** $S_{\text{Ssk1\_[(D544)]-\{p\}}}$) **AND** ($S_{\text{Ssk1\_[RR]--0}}$ **OR** $S_{\text{Ssk1\_[RR]--Ssk2\_[ssk1]}}$)) AND $S_{\text{Ssk2\_[ssk1]--0}}$ **OR** $S_{\text{Ssk1\_[RR]--Ssk2\_[ssk1]}}$) AND (($R_{\text{Ssk1\_[RR]\_ppi+\_Ssk2\_[ssk1]}}$ AND ($S_{\text{Ssk1\_[RR]--0}}$ AND $S_{\text{Ssk2\_[ssk1]--0}}$)) OR ($S_{\text{Ssk1\_[RR]--Ssk2\_[ssk1]}}$ AND NOT ($R_{\text{Ssk1\_[RR]\_ppi-\_Ssk2\_[ssk1]}}$ AND $S_{\text{Ssk1\_[RR]--Ssk2\_[ssk1]}}$)))

These are the final update rules in the ansatz update rules.

Eq. 9. Smoothing:

$$R''_l(t) = R_l(t) \bigcap_{\substack{S_j\ consumed \\ by\ R_l}} S_j(t) \cup \quad S_j(t+1)$$

The smoothing generates a term in which the reaction fires if the reaction (regulatory state) is true AND the substrate either is true OR becomes true in the following time step. We exemplify with two reaction terms, with R' included for comparison:

Before:

$R'_{\text{Sln1\_ap+\_Sln1\_[(Site)]}}$ = $R_{\text{Sln1\_ap+\_Sln1\_[(Site)]}}$ AND $S_{\text{Sln1\_[(Site)]-\{0\}}}$

After:

$R''_{\text{Sln1\_ap+\_Sln1\_[(Site)]}}$ = $R_{\text{Sln1\_ap+\_Sln1\_[(Site)]}}$ AND ($S_{\text{Sln1\_[(Site)]-\{0\}}}$ OR (C_{Sln1} AND ($R'_{\text{Sln1\_[(Site)]\_pt\_Ypd1\_[(H64)]}}$ OR ($S_{\text{Sln1\_[(Site)]-\{0\}}}$ AND NOT $R'_{\text{Sln1\_ap+\_Sln1\_[(Site)]}}$))))

i.e.:

$R''_{\text{Sln1\_ap+\_Sln1\_[(Site)]}}$ = $R_{\text{Sln1\_ap+\_Sln1\_[(Site)]}}$ AND ($S_{\text{Sln1\_[(Site)]-\{0\}}}$ OR (($S_{\text{Sln1\_[(Site)]-\{0\}}}$ **OR** $S_{\text{Sln1\_[(Site)]-\{p\}}}$) AND (($R_{\text{Sln1\_[(Site)]\_pt\_Ypd1\_[(H64)]}}$ AND ($S_{\text{Sln1\_[(Site)]-\{p\}}}$ AND $S_{\text{Ypd1\_[(H64)]-\{0\}}}$)) OR ($S_{\text{Sln1\_[(Site)]-\{0\}}}$ AND NOT ($R_{\text{Sln1\_ap+\_Sln1\_[(Site)]}}$ AND $S_{\text{Sln1\_[(Site)]-\{0\}}}$))))

Before:

$R'_{\text{Ssk1\_[RR]\_ppi-\_Ssk2\_[ssk1]}}$ = $R_{\text{Ssk1\_[RR]\_ppi-\_Ssk2\_[ssk1]}}$ AND $S_{\text{Ssk1\_[RR]--Ssk2\_[ssk1]}}$

After:

$R''_{\text{Ssk1\_[RR]\_ppi-\_Ssk2\_[ssk1]}}$ = $R_{\text{Ssk1\_[RR]\_ppi-\_Ssk2\_[ssk1]}}$ AND ($S_{\text{Ssk1\_[RR]--Ssk2\_[ssk1]}}$ OR ((C_{Ssk1} AND C_{Ssk2}) AND ($R'_{\text{Ssk1\_[RR]\_ppi+\_Ssk2\_[ssk1]}}$ OR ($S_{\text{Ssk1\_[RR]--Ssk2\_[ssk1]}}$ AND NOT $R'_{\text{Ssk1\_[RR]\_ppi-\_Ssk2\_[ssk1]}}$))))

i.e.:

$R''_{\text{Ssk1\_[RR]\_ppi-\_Ssk2\_[ssk1]}}$ = $R_{\text{Ssk1\_[RR]\_ppi-\_Ssk2\_[ssk1]}}$ AND ($S_{\text{Ssk1\_[RR]--Ssk2\_[ssk1]}}$ OR (((($S_{\text{Ssk1\_[(D544)]-\{0\}}}$ **OR** $S_{\text{Ssk1\_[(D544)]-\{p\}}}$) **AND** ($S_{\text{Ssk1\_[RR]--0}}$ **OR** $S_{\text{Ssk1\_[RR]--Ssk2\_[ssk1]}}$)) AND $S_{\text{Ssk2\_[ssk1]--0}}$ **OR** $S_{\text{Ssk1\_[RR]--Ssk2\_[ssk1]}}$) AND (($R_{\text{Ssk1\_[RR]\_ppi+\_Ssk2\_[ssk1]}}$ AND ($S_{\text{Ssk1\_[RR]--0}}$ AND $S_{\text{Ssk2\_[ssk1]--0}}$)) OR ($S_{\text{Ssk1\_[RR]--Ssk2\_[ssk1]}}$ AND NOT ($R_{\text{Ssk1\_[RR]\_ppi-\_Ssk2\_[ssk1]}}$ AND $S_{\text{Ssk1\_[RR]--Ssk2\_[ssk1]}}$)))))

These terms are then used to replace the R' in the production terms in equation 8. We exemplify with a single state update:

$S_{\text{Sln1\_[(Site)]-\{p\}}}$ = C_{Sln1} AND ($R'_{\text{Sln1\_ap+\_Sln1\_[(Site)]}}$ OR ($S_{\text{Sln1\_[(Site)]-\{p\}}}$ AND NOT $R'_{\text{Sln1\_[(Site)]\_pt\_Ypd1\_[(H64)]}}$))

becomes:

$S_{\text{Sln1\_[(Site)]-\{p\}}}$ = C_{Sln1} AND ($R''_{\text{Sln1\_ap+\_Sln1\_[(Site)]}}$ OR ($S_{\text{Sln1\_[(Site)]-\{p\}}}$ AND NOT $R'_{\text{Sln1\_[(Site)]\_pt\_Ypd1\_[(H64)]}}$))

i.e.:

$S_{\text{Sln1\_[(Site)]-\{p\}}}$ = ($S_{\text{Sln1\_[(Site)]-\{0\}}}$ **OR** $S_{\text{Sln1\_[(Site)]-\{p\}}}$) AND ($R_{\text{Sln1\_ap+\_Sln1\_[(Site)]}}$ AND ($S_{\text{Sln1\_[(Site)]-\{0\}}}$ OR (($S_{\text{Sln1\_[(Site)]-\{0\}}}$ **OR** $S_{\text{Sln1\_[(Site)]-\{p\}}}$) AND (($R_{\text{Sln1\_[(Site)]\_pt\_Ypd1\_[(H64)]}}$ AND ($S_{\text{Sln1\_[(Site)]-\{p\}}}$ AND $S_{\text{Ypd1\_[(H64)]-\{0\}}}$)) OR ($S_{\text{Sln1\_[(Site)]-\{0\}}}$

AND NOT ($R_{Sln1\_ap+\_Sln1\_[(Site)]}$ AND $S_{Sln1\_[(Site)]\text{-}\{0\}}$)))) OR ($S_{Sln1\_[(Site)]\text{-}\{p\}}$ AND NOT $R_{Sln1\_[(Site)]\_pt\_Ypd1\_[(H64)]}$ AND ($S_{Sln1\_[(Site)]\text{-}\{p\}}$ AND $S_{Ypd1\_[(H64)]\text{-}\{0\}}$)))
